# A Comprehensive Proteomics Analysis of the JC Virus (JCV) Large and Small Tumor Antigen Interacting Proteins: Large T Primarily Targets the Host Protein Complexes with V-ATPase and Ubiquitin Ligase Activities While Small t Mostly Associates with Those Having Phosphatase and Chromatin-Remodeling Functions

**DOI:** 10.3390/v12101192

**Published:** 2020-10-20

**Authors:** Sami Saribas, Mahmut Safak

**Affiliations:** Department of Neuroscience, Lewis Katz School of Medicine at Temple University, 3500 N. Broad Street, Philadelphia, PA 19140, USA; saribas@temple.edu

**Keywords:** large T antigen, small t antigen, interactome, transformation, chromatin remodeling, tumorigenesis, progressive multifocal leukoencephalopathy, polyomavirus, v-ATPAse, PPP4, Smc5/6, ubiquitin E3 ligase, PP2A, SDHA, SDHB, Smarca5, JCV, SV40, BKV, Merkel cell carcinoma

## Abstract

The oncogenic potential of both the polyomavirus large (LT-Ag) and small (Sm t-Ag) tumor antigens has been previously demonstrated in both tissue culture and animal models. Even the contribution of the MCPyV tumor antigens to the development of an aggressive human skin cancer, Merkel cell carcinoma, has been recently established. To date, the known primary targets of these tumor antigens include several tumor suppressors such as pRb, p53, and PP2A. However, a comprehensive list of the host proteins targeted by these proteins remains largely unknown. Here, we report the first interactome of JCV LT-Ag and Sm t-Ag by employing two independent “affinity purification/mass spectroscopy” (AP/MS) assays. The proteomics data identified novel targets for both tumor antigens while confirming some of the previously reported interactions. LT-Ag was found to primarily target the protein complexes with ATPase (v-ATPase and Smc5/6 complex), phosphatase (PP4 and PP1), and ligase (E3-ubiquitin) activities. In contrast, the major targets of Sm t-Ag were identified as Smarca1/6, AIFM1, SdhA/B, PP2A, and p53. The interactions between “LT-Ag and SdhB”, “Sm t-Ag and Smarca5”, and “Sm t-Ag and SDH” were further validated by biochemical assays. Interestingly, perturbations in some of the LT-Ag and Sm t-Ag targets identified in this study were previously shown to be associated with oncogenesis, suggesting new roles for both tumor antigens in novel oncogenic pathways. This comprehensive data establishes new foundations to further unravel the new roles for JCV tumor antigens in oncogenesis and the viral life cycle.

## 1. Introduction

Viruses are all obligatory intracellular parasites due to their limited genomic coding capacity. A number of proteins encoded by their small genomes are insufficient to form a host-independent organism. Various viral proteins were shown to target many different host proteins and pathways in order to create a more conducive environment for the successful propagation of the invader in host cells [1,2]. However, in the absence or limited viral replication cycle, some viral proteins gain an opportunity to alter the host cells, leading to transformation [3].

The most extensively studied DNA tumor virus groups are comprised of three families with small genome, which include polyomaviruses, papillomaviruses, and adenoviruses [4]. We have learned much about the mechanisms of the cell transformation by studying the oncogenic properties of various viral proteins encoded by these viruses [4,5]. The first member among these tumor viruses was isolated from rabbits by Richard Shope during the early 1930s and classified under papillomavirus family [6,7]. The second (mouse polyomavirus) [8] and the third (simian virus-40 (SV40)) [9] oncogenic viruses were isolated from the mouse-derived leukemic extracts and the monkey renal culture cells, respectively. These last two oncogenic viruses were also of animal origin and classified under a new virus family, polyomaviruses, during the early 1950s and 1960s, respectively. However, the first two human polyomaviruses (JC virus (JCV) and BK virus (BKV)) were discovered approximately 11 years later after the discovery of SV40 [10,11]. JCV is known to cause a deadly brain disease, progressive multifocal leukoencephalopathy [10], whereas BKV is the etiologic agent of a kidney disease, polyomavirus-associated nephropathy [11,12]. The discovery of additional human polyomaviruses has been recently facilitated due to availability of the new technologies, including the rolling circle amplification and digital transcriptome subtraction. The current number of human polyomavirus has now risen to 14 [13,14]. Note that not every isolated human polyomavirus was proven to be associated with any particular human disease. Besides JCV and BKV, only trichodysplasia spinulosa-associated polyomavirus (TSPyV), and Merkel cell carcinoma-associated polyomavirus (MCPyV) were, to date, reported to cause human diseases, such as trichodysplasia spinulosa [15] and Merkel cell carcinoma [16], respectively.

Cancer is a multifactorial disease and manifests itself when the balance between growth- promoting and growth-inhibitory signals is disrupted [17]. This disruption primarily occurs when the key components of the cell regulatory pathways are functionally altered either by mutations at the gene level or by the viral-encoded oncoproteins. One of the unifying characteristics of the abovementioned small tumor viruses is to encode various oncogenic proteins with the ability to target and alter the specific functions of numerous cell regulatory pathways [4,14,18,19,20,21]. For example, the adenovirus E1A protein was first demonstrated to target and inhibit one of the key cell cycle regulators of the host cells, pRb, [22], which regulates the G1-to-S phase transition [23]. In the same year, the interaction and, thereby, inhibition of the regulatory functions of the same protein (pRb) by another viral oncogenic protein, SV40 large T antigen (LT-Ag), was also demonstrated by DiCaprio et al. [24] from a different tumor virus family (polyomavirus). In contrast to these customary inhibitory functions by adenovirus E1A and SV40 LT-Ag when they bind to pRb, several year later Gonzalez at al. (2001) reported an interesting finding regarding function of a new oncogenic viral protein, the human papillomavirus E7, where it was found to lead to the degradation of pRb rather than stabilizing and, thereby, inhibiting its functions. These findings stress the fact that the different viral oncoproteins can inhibit the same host protein by different mechanisms. 

Over time, it was also clear that the various oncogenic viral proteins from these three viral groups could differentially target and regulate the fate of specific host proteins (stabilization or degradation). For instance, SV40 LT-Ag targets and stabilizes one of the most important stress-inducible cell cycle regulators of the host cells, p53. This was first reported during the late 1970s [25,26]. Under normal conditions, p53 arrests cells at the G0 and G2 phases of the cell cycle depending on the stress type inflicted on the cells [27]. Later mapping studies revealed that the interaction of LT-Ag with p53 occurs through the DNA binding domain of p53 [28,29] and this interaction blocks DNA binding activity of p53 to the p53-responsive gene promoters and, thus, inhibits the p53-mediated gene expression and, therefore, p53-dependent cell cycle arrest [30]. Following this discovery, the stabilization and inhibition of p53 by another oncogenic viral protein, adenovirus E1B 55k protein, was also reported by Sarnow et al. [31]. In contrast to these findings, the targeting of p53 by another oncogenic viral protein, human papillomavirus (HPV) E6 protein, did not yield the stabilization but the degradation of the protein via the ubiquitin pathway [32,33,34,35,36,37].

Since the discovery of the first polyomavirus (mouse polyomavirus), the oncogenic potential of various polyomaviruses has been tested in both various cell cultures and animal model systems, based on the fact that this group of viruses all primarily encode two oncogenic proteins, LT-Ag and Sm t-Ag. The primary transforming protein encoded by the mouse polyomavirus is, however, the middle T antigen [38]. LT-Ag plays a major role in cell transformation in the experimental animals including mice and hamsters but, in human cells, it requires the additional perturbation by Sm t-Ag in order to complete the transformation process [39,40]. The MCPyV Sm t-Ag, however, appears to be more aggressive than LT-Ag in its oncogenicity [41,42]. Mechanistically, the transforming activity of LT-Ag of most of the human polyomaviruses appears to be, at least in part, mediated by its interaction with two important tumor suppressor proteins, p53 and pRb [43,44,45]. The small t antigen, on the other hand, primarily targets PP2A in cells [43,46,47,48]. Among all human polyomaviruses, MCPyV is currently the only polyomavirus linked to the development of the specific human tumors, Merkel cell carcinoma [16], which is a rare neuroendocrine cutaneous malignancy [49,50]. A major requirement for the survival of the MCPyV-containing carcinoma cells is to maintain the expression of both LT-Ag and Sm t-Ag. In other words, the silencing of the expression of both proteins causes the cell death [51]. Another interesting feature of these cells is that viral genome is integrated into the host genome where the C-terminus of the LT-Ag was found to be truncated but the N-terminal domain along with the LXCXE, the pRb binding motif, remains intact. The DNA binding, helicase, and cell-growth inhibitory domains are lost [52,53]. 

JCV LT-Ag and Sm t-Ag are also known to induce cell transformation in tissue culture [54] as well as in experiment animals [55,56,57,58,59,60,61]. In addition, the detection of JCV DNA in various human tumors [62,63,64,65] suggests that this virus may also play roles in development of some human tumors similar to MCPyV. As discussed above, all oncogenic viral proteins, in one way or another, perturb various cellular pathways or target various host proteins. This targeting may result in either inhibition or promotion of the activity of their targets to initiate the oncogenic process. Although a number of studies have previously revealed several targets of JCV LT-Ag and Sm t-Ag, the complete maps of their interactome remain incomplete. Thus, through the implementation of a comprehensive proteomics approach, we determined the complete interactomic maps for both proteins by employing an elegant affinity purification (AP) system followed by mass spectroscopy (MS). Two independent data runs yielded 622 targets in total for both LT-Ag and Sm t-Ag. After eliminating backgrounds based on the criteria stated in the text, the 139 targets were selected for both proteins, 68 for LT-Ag and 71 for Sm t-Ag. Among them, 27 targets are common targets by both proteins. The remaining 41 and 44 interactions were unique to LT-Ag and Sm t-Ag, respectively. The selected targets were further analyzed by employing two different web tools: (1) “FunRich Software”, a functional enrichment analysis tool (http://www.funrich.org) to categorize the interacting proteins into various groups [66,67] and (2) a “STRING database” (Search Tool for the Retrieval of Interacting Genes/Proteins), a comprehensive protein–protein interaction network-building web resource (https://string-db.org).

The proteomics data revealed that JCV LT-Ag primarily interacts with proteins’ complexes with ATPase activities (v-ATPase and Smc5/6 complex) and phosphatase activities (PP4 and PP1). LT-Ag also interacts with the components of protein degradation network such as E3-ubiquitin ligase. Sm t-Ag mainly targets phosphatases such as PP2A and protein assemblies involved in the chromatin remodeling such as Smarca5. JCV Sm t-Ag also interacts with the protein transport network, chaperones, heterogeneous ribonuclear proteins, and mitochondrial proteins such as succinate dehydrogenase. Our data showed that both tumor antigens also target common protein complexes such as actin/myosin network and ribosomal proteins. Collectively, this work presents the first comprehensive proteomics data analysis for JCV tumor antigens, LT-Ag and Sm t-Ag, and establishes a new foundation to further unravel the critical regulatory roles of both proteins in cell transformation and JCV life cycle.

## 2. Materials and Methods 

### 2.1. Construction of a T7-2xStrep Affinity Purification System

Creation of a “Twin Strep Tag” and use of Strep-Tactin affinity purification system were previously reported (Twin-Strep Tag: GG-SA-WNHPQFEK-GGGSGSGG-SA-WSHPQFEK-GS) [68]. We further modified this cassette to enhance the expression of JCV LT-Ag in cells by fusing the T7 tag to the N-terminus of the Twin Strep Tag, (MASRMASMTGGQQM-GG-SA-WNHPQFEK-GGGSGSGG-SA-WSHPQFEK-GS) and named “T7-2xStrep Tag”, where the T7 tag portion is underlined. This modified tag was recently subcloned into the pcDNA3.1 (+) vector at *Hind*III/*Bam*HI restriction sites, as previously described [2], and designated as pcDNA3.1 (+)-T7-2xStrep. This T7-2xStrep Tag in this vector was successfully used in JCV agnoprotein proteomics studies, as described [2]. In this study, JCV LT-Ag and JCV Sm t-Ag coding sequences were also subcloned into the same vector (pcDNA3.1 (+)-T7-2xStrep) at *Eco*RI/*Xho*I and *Bam*HI/*Eco*RI restriction sites in frame with T7-2xStrep-tag at their N-terminus, respectively. The resulting plasmids were designated as pcDNA3.1 (+)-T7-2xStrep-JCV LT-Ag and pcDNA3.1 (+)-T7-2xStrep-JCV Sm t-Ag, respectively. For a control vector, pcDNA3.1 (+)-T7-2xStrep-Stop-JCVAgno was used. In this plasmid, Agno expression was blocked by a stop codon, but the expression of theT7-2xStrep tag was allowed. The construction of pcDNA3.1 (+)-T7-2xStrep-JCVAgno and its stop codon mutant was described previously [2].

### 2.2. Cell Lines

SVG-A is a subclone of the human cell line established by transforming primary human fetal glial cells with an origin-defective SV40 mutant [69]. HEK293T cells (ATCC, catalog no. CRL-3216) are the human embryonic kidney cell lines. Both cell lines were grown in Dulbecco’s Modified Eagle’s Medium (DMEM) (ThermoFisher, Waltham, MA, USA, catalog no. 31600-034) supplemented with 10% heat-inactivated fetal bovine serum (FBS) (Gemini, West Sacramento, CA, USA, catalog no 100-106) and antibiotics (penicillin-streptomycin (100 µg/mL) (Gemini, West Sacramento, CA, USA, catalog no 400-1001) and ciprofloxacin (MP biochemical, Irvine, CA, USA, catalog no. 199020, 10 µg/mL)). Cells were maintained at 37 °C in a humidified atmosphere and supplemented with 7% CO_2_.

### 2.3. Peptide Synthesis 

“T7-2xStrep peptide” (MASRMASMTGGQQM-GG-SA-WNHPQFEK-GGGSGSGG-SA-WSHPQFEK-GS) was commercially synthesized by Biosynthesis company (Lewisville, TX, USA, https://www.biosyn.com), as previously described [2].

### 2.4. Expression of the T7-2xStrep-JCV LT-Ag and T7-2xStrep-JCV Sm t-Ag

HEK293T cells were grown into ~80% confluency in tissue culture plates (100 mm in diameter, Becton Dickinson, Franklin lakes, NJ, USA, catalog no. 353002) treated with Poly-L-lysine (Sigma, St. Louis, MO, USA, catalog no. p8920-100 mL) in DMEM, supplemented with 10% fetal bovine serum (FBS) and antibiotics (penicillin/streptomycin, ThermoFisher, Waltham, MA, USA, catalog no. 15070063). The plated HEK293T cells were transfected with either pcDNA3.1 (+)-T7-2xStrep-JCV LT-Ag (experimental), pcDNA3.1 (+)-T7-2xStrep-JCV Sm t-Ag (experimental) or pcDNA3.1 (+)-T7-2xStrep-Stop-JCVAgno (control) expression plasmids (30 µg each) using calcium-phosphate precipitation method [70]. At 24 h post-transfection, whole-cell extracts were prepared and tested for the expression of the protein of interest by Western blotting, as described below.

### 2.5. Affinity Purification of the T7-2xStrep-JCV LT-Ag- and T7-2xStrep-JCV Sm t-Ag-Associated Proteins

At 24 h post-transfection, whole-cell extracts (WCE) were prepared from the transfected and untransfected HEK293T cells. Briefly, cells were washed with 1× PBS twice, lysed in 2 mL of TNN buffer (50 mM Tris-HCl (pH 7.4), 150 mM NaCl, 1 mM EDTA, and 1.0% NP-40 in the presence of protease inhibitors (Sigma, St. Louis, MO, USA, catalog no. P8340)), collected, and incubated at 4 °C on a rocking platform for 30 min. The cell lysates were then cleared by centrifugation at 17,000× *g* for 10 min at 4 °C. The NP-40 concentration in the whole-cell extracts (control and experimental) were adjusted to 0.3% (30 milligram total protein) and incubated with 150 µL of MagStrep “Type 3” XT magnetic beads (IBA Lifesciences, Göttingen, Germany, catalog no. 2-4090-002) at 4 °C for 16 h on a racking platform to capture T7-2xStrep-tagged LT-Ag and Sm t-Ag along with LT-Ag- and Sm t-Ag-bound proteins. As a control, 1 µg of T7-2xStrep peptide was also incubated with “the control extract” during the protein purification steps. LT-Ag- and Sm t-Ag-interacting protein complexes were then washed in TNN buffer containing 0.3% NP40 by using a bead-capturing magnet system (“DynaMag”, ThermoFisher, Waltham, MA, USA, catalog no. 12321D) and eluted in the same buffer containing 50 mM biotin for proteomics studies. This affinity purification of the LT-Ag- and Sm t-Ag-interacting proteins was repeated using extracts prepared from other independently transfected cells.

### 2.6. Western Blotting, Silver, and Colloidal Blue Staining

Thirty milligrams of WCE prepared from either transfected (experimental) or control (transfected only with control vector) cells were incubated with MagStrep type 3 XT magnetic beads (150 µL) for 16 h at 4 °C on a racking platform for affinity purification of either LT-Ag- or Sm t-Ag-binding proteins. The bead–protein complexes were then washed with a washing buffer (50 mM Tris-HCl (pH 7.4), 150 mM NaCl, 1 mM EDTA, and 0.3% NP-40) and split into three equal fractions. One fraction was resolved on a “NUPAGE 4–12% Bis-Tris protein gel” (Invitrogen, catalog no. NP0337Box) using MES-SDS buffer (Invitrogen, catalog no. NP0002) and analyzed by Western blotting using an α-T7 antibody (Novagen, Madison, WI, USA, catalog no. 69522-3). The protein complexes from the other two fractions were eluted with biotin, as described in manufacturers’ recommendations, and separated on two different “NUPAGE 4–12% Bis-Tris protein gels” using MES-SDS buffer followed by either silver staining (ThermoFisher, Waltham, MA, catalog no. 24600) or colloidal blue staining (ThermoFisher, Waltham, MA, USA, catalog no. LC6025) procedures to visualize the samples.

### 2.7. LC-MS/MS Analyses and Data Processing

Liquid chromatography tandem mass spectrometry (LC-MS/MS) analysis was performed by the Proteomics and Metabolomics Facility at the Wistar Institute, Philadelphia, PA, using a Q Executive HF mass spectrometer (ThermoFisher, Waltham, MA, USA) coupled with a Nano-ACQUITY UPLC system (Waters). Samples were digested in gel with trypsin and injected onto a UPLC Symmetry trap column (180 μm i.d. × 2 cm packed with 5 μm C18 resin) (Waters, Milford, MA, USA). Tryptic peptides were separated by reversed phase HPLC on a BEH C18 Nanocapillary analytical column (75 μm i.d. × 25 cm, 1.7 μm particle size) (Waters, Milford, MA, USA) using a 95-min gradient formed by solvent A (0.1% formic acid in water) and solvent B (0.1% formic acid in acetonitrile). A 30-min blank gradient was run between sample injections to minimize carryover. Eluted peptides were analyzed by the mass spectrometer set to repetitively scan m/z from 400 to 2000 in positive ion mode. The full MS scan was collected at 60,000 resolution followed by data-dependent MS/MS scans at 15,000 resolution on the 20 most-abundant ions exceeding a minimum threshold of 10,000. Peptide match was set as preferred; exclude isotopes’ option and charge-state screening was enabled to reject unassigned charged ions. 

### 2.8. Proteomics Data Analysis Using “STRING Database” and “FunRich” Software

Proteomics data were obtained after mass spectroscopy (MS) analysis of two independently run affinity purifications (AP) that contained the JCV LT-Ag- and Sm t-Ag-interacting proteins. Proteomics analysis allowed us to generate a list of JCV LT-Ag- and Sm t-Ag-interacting proteins with a minimum of two significant peptides with no background based on the combination of two AP/MS runs. Note that, in a few cases, one significant peptide and low background were also included. The proteomics data were analyzed using the “STRING database”, which is a comprehensive protein–protein interaction database (https://string-db.org). Two independent sets of AP/MS data compiled as a list of proteins, which interact with either JCV LT-Ag or Sm t-Ag without background, were used as an input into the “STRING program” to generate a number of protein-protein interaction networks that both tumor antigens target. 

We also analyzed the AP/MS data using the “FunRich software program, a standalone software program” [66,67]. This program analyzes the proteomics data based on the different categories such as cellular components, molecular functions, biological processes, biological pathways, protein domains, site of expressions, transcription factors, and clinical phenotypes. 

### 2.9. GST Pull-Down Assays

The creation of the fusion protein of glutathione-s-transferase (GST) with JCV LT-Ag full-length (aa 1-688) and with the several-deletion mutants of JCV LT-Ag was previously described [71], where JCV LT-Ag and its deletion mutants were cloned in pGEX2T vector at the *Eco*RI restriction site. The creation of GST with the full-length JCV Sm t-Ag was also previously described [72], where the full-length JCV Sm t-Ag had been cloned into the *Bam*HI/*Eco*RI restriction sites. The following plasmids were used for GST pull-down assays in this study: pGEX2T (empty vector, GE Healthcare, Chicago, IL, USA), pGEX2T-JCV LT-Ag (1–688), pGEX2T-JCV LT-Ag (1–411) pGEX2T-JCV LT-Ag (1–265), pGEX2T-JCV LT-Ag (1–82), pGEX2T-JCV LT-Ag (266–688), pGEX2T-JCV LT-Ag (412–688), and pGEX2T-JCV Sm t-Ag (1–172). The expression and purification of the GST-tagged proteins were described previously [73]. Two micrograms of either GST alone or GST-JCV LT-Ag (aa 1–688, full-length), or GST-JCV LT-Ag deletion mutants as indicated above were immobilized on glutathione-4B Sepharose beads (*Glutathione Sepharose*^TM^ 4B, GE healthcare, catalog no. 17-0756-01) and incubated with 0.5 mg of whole-cell extract prepared from HEK293T cells transfected with pcDNA3.1-Smc6-flag plasmid (GenScript, catalog number: OHu04128D) at 4 °C overnight in lysis buffer containing 50 mM Tris-HCl (pH 7.4), 150 mM NaCl, and 0.5% NP-40 and supplemented with a cocktail of proteinase inhibitors (Sigma, catalog no. P 8340-5ml). Smc6 stands for “structural maintenance of chromosomes protein 6”. Protein complexes formed between LT-Ag and cellular proteins were washed extensively with lysis buffer, resolved on a SDS-8% PAGE, and analyzed by Western blotting where membranes were probed with the primary mouse monoclonal α-flag antibody (Invitrogen, catalog no. MA1-91878) directed against the flag-tag of Smc6 for 6 h and washed by Tris-buffered saline-tween buffer (TBST) (50 mM Tris-HCL pH 7.6, 150 mM NaCl, 0.1% Tween-20) three times (10 min each). Then membranes were incubated with secondary goat α-mouse IRDye 680LT (LI-COR, catalog no. 926–68070) antibody for 45 min, washed twice with TBST (10 min each), and scanned using Odyssey^®^ CLx Infrared Imaging System (LI-COR) to detect the protein of interest. 

In addition, 2 µg of either GST alone or GST-JCV Sm t-Ag (aa 1–172, full-length), immobilized on glutathione-4B Sepharose beads was incubated with 0.5 mg of whole-cell extract prepared from HEK293T cells transfected with either pcDNA3.1-SDHB-flag plasmid (GenScript, Piscataway, NJ, USA, catalog number: OHu18105D) or pcDNA3.1-Smarca5-flag plasmid (GenScript, Piscataway, NJ, USA, catalog number: OHu12497D) at 4 °C overnight in lysis buffer. After extensively washing with lysis buffer, the bound proteins were either resolved on SDS-15% PAGE (for SDHB pull down) or on a SDS-8%-PAGE (for Smarca5 pull down) and analyzed by Western blotting using an α-flag antibody. Smarca stands for “SWI/SNF-related, matrix-associated, actin-dependent regulator of chromatin, subfamily A, member 5”. SDHB stands for “succinate dehydrogenase complex iron sulfur subunit B”. Note that the whole-cell extracts prepared from untransfected HEK293T cells were used as negative controls in the GST pull-down assays. 

### 2.10. Immunocytochemistry (ICC)

SVG-A cells were plated onto 60-mm tissue culture plates (Falcon, catalog no. 353002), grown to ~80% confluency, and transfected with the following expression plasmid combinations: Either with pcDNA 3.1(+) T7-2xStrep-JCV LT-Ag plus pcDNA 3.1 (+)-Smc6-flag or pCGT7-JCV Sm t-Ag plus pcDNA 3.1 (+)-Smarca5 combinations using lipofectamine 3000 reagent (Invitrogen, catalog no. L3000008). At 16 h post-transfection, cells were washed with 1× PBS, transferred onto glass-slide chambers (Nunc, catalog no. 154461), and incubated for an additional 24 h. Next, the cells were washed twice with 1× PBS, fixed in cold acetone for 2 min, washed twice with 1× PBS, and incubated with 5% bovine serum albumin prepared in 1× PBS for 2 h. Chamber slides were then incubated with a combination of α-T7 polyclonal (Genescript, Piscataway, NJ, USA, catalog no. A00622) (1:200 dilution) and α-FLAG monoclonal (Invitrogen, catalog no. MA1-91878) antibodies overnight. Cells were then washed three times with TBST buffer for 10-min intervals and subsequently incubated either with a fluorescein isothiocyanate (FITC)-conjugated goat α-rabbit (Abcam, Cambridge, MA, USA, catalog no. Ab6717) or Rhodamine-conjugated goat α-mouse (MilliporeSigma, Burlington, MA, USA, catalog no. AP124R) secondary antibodies for 45 min. Cells were then washed with TBST buffer three times for 10 min each and incubated with DAPI (4′,6-Diamidino-2-Phenylindole, Dihydrochloride) (ThermoFisher, Waltham, MA, USA, catalog no. D1306) (300 ng/mL prepared in 1× PBS) to stain the nucleus. The cover glass was mounted onto the slide chambers using “ProLong^®^ Gold Antifade” mounting medium (ThermoFisher, Waltham, MA, USA, catalog no. P36934) and dried overnight. Slides were then examined under a fluorescence microscope (Leica, Morrisville, NC, USA, DMI-6000B, objective: HCX PL APD 60×/1.25 oil, employing LAS AF operating software) to examine the proteins of interest. In addition, the cellular distribution profiles of both LT-Ag and Sm t-Ag were also analyzed by ICC in SVG-A cells, as described under their respective figure legends. 

## 3. Results and Discussion

### 3.1. The Use of a T7-2xStrep Tag and Magnetic Bead Affinity Purification System to Pull down the JCV LT-Ag- and JCV Sm t-Ag-Interacting Proteins

Short peptide affinity tags are widely used for a number of applications in molecular biology for affinity purification of the protein of interest and for analyzing the distribution patterns of a protein by immunocytochemistry if a specific antibody for a particular protein of interest is not available.

**Figure 1:**
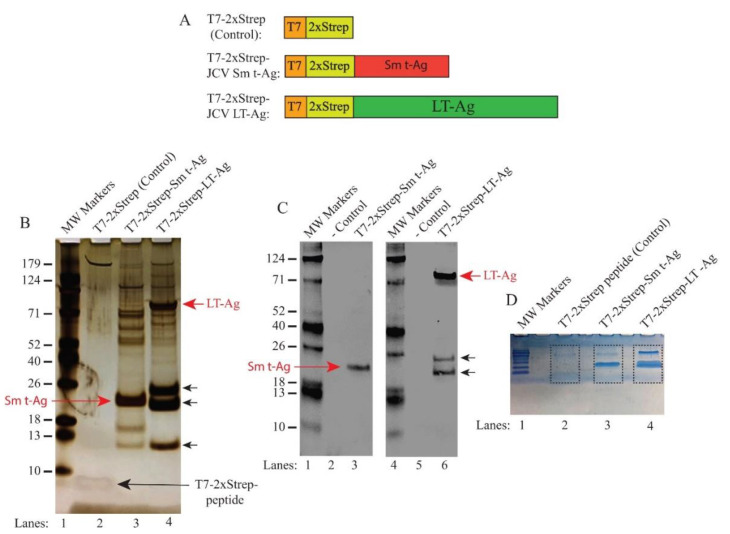
Analysis of the JCV LT-Ag- and JCV Sm t-Ag-associated host proteins by silver and colloidal blue staining. (**A**) Graphical representation of the T7-2xStrep affinity purification system. (Upper panel) The T7-2xStrep sequences were cloned into the *Hind*III/*Bam*HI restriction sites of pcDNA3.1 (+) vector, as previously described [2]. (Middle panel) JCV Sm t-Ag coding sequences were then cloned into the *Bam*HI/*Xho*I restriction sites in frame with the T7-2xStrep tag, as described in Materials and Methods. (Lower panel) JCV LT-Ag coding sequences (with no intron) were then cloned into the *Eco*RI/*Xho*I restriction sites in frame with the T7-2xStrep tag, as described in Materials and Methods. (**B**) Analysis of the JCV LT-Ag- and Sm t-Ag-associated proteins by silver staining. HEK293T cells were transfected with either pcDNA3.1(+)-T7-2xStrep-Stop-Agno (control, expresses only T7-2xStrep tag) (lane 2), pcDNA3.1(+)-T7-2xStrep-JCV Sm t-Ag (lane 3), or pcDNA3.1(+)-T7-2xStrep-JCV LT-Ag (lane 4) plasmids. The whole-cell extracts (10 mg) prepared from these transfectants were then subjected to affinity purification using MagStrep “Type 3” XT magnetic beads. After elution with biotin, protein samples were resolved on a NUPAGE 4–12% Bis-Tris protein gel and stained with silver staining reagents, as described in the Materials and Methods section. Note that 1 µg of a synthetic T7-2xStrep peptide was also incubated along with the extracts prepared from the control cells to demonstrate the nonspecific binding to the tag alone (lane 2). Note that control extracts were also incubated with a control peptide (T7-2xStrep) in order to subtract out the nonspecific binding proteins to T7-2xStrep to obtain a reliable and low background in our proteomics studies. The migration patterns of JCV LT-Ag and Sm t-Ag are indicated by the arrows. The unlabeled arrows point to the alternatively spliced forms of JCV LT-Ag [74,75]. (**C**) Western blot analysis of the affinity-purified JCV Sm t-Ag and JCV Sm t-Ag-interacting proteins; and JCV LT-Ag and JCV LT-Ag-interacting proteins. In parallel to the experiments described in panel B, the affinity-purified protein samples were analyzed by Western blotting using α-T7 antibody. On each lane, the affinity purified whole-cell extracts (20 µL/lane) were loaded on a NUPAGE 4–12% Bis-Tris protein gel as indicated. MW: Molecular weight. (**D**) In parallel to the protocols described for panel B, 10 mg of whole-cell extract prepared from the HEK293T cells transfected with either control plasmid plus incubated with T7-2xStrep peptide (lane 2) or transfected with the experimental plasmids (lanes 3 and 4, as indicated) were affinity purified and resolved shortly on a NUPAGE 4–12% gradient gel and stained with colloidal blue. Then, the encased bands by the dash-lined rectangles were excised from the gel and analyzed by LC-MS/MS after in gel digestion with trypsin.

**Figure 2:**
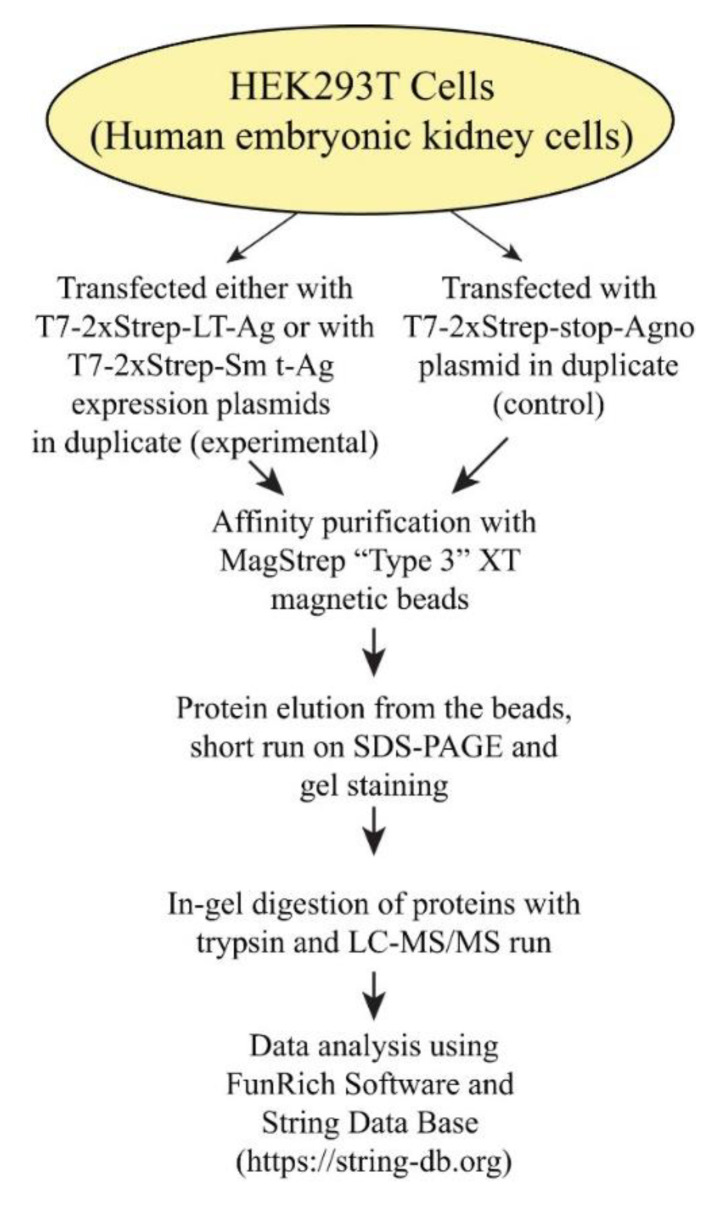
A graphical presentation of the experimental design to determine the JCV LT-Ag- and JCV Sm t-Ag-interacting proteins by affinity purification/mass spectroscopy analysis (AP/MS).

One of the desirable features of these tags is to provide highly pure and functional proteins at physiological conditions after a rapid and one-step elution. For example, the “Strep tag II” harbors such a convenient feature and selectively binds to its commercially available target, “Strep Tactin^®^”, an engineered streptavidin protein. Recently, a more efficient system with a higher affinity was developed and designated as “Twin Strep Tag II”. This new “Twin Strep Tag” system has a ~100-fold increased affinity to its target, Strep-Tactin XT, which is linked to magnetic beads. The “Strep-Tag II” has several advantages over several other short peptide affinity tags, including Flag (DYKDDDDK) and 6xHis (6xHHHHHH). These advantages include being biologically inert, proteolytically stable, and not interfering with membrane translocation or protein folding. Therefore, we believe that this tag does not interfere with either LT-Ag or Sm t-Ag functions in cells. More importantly, Strep-tagged proteins can be eluted with biotin under physiological and mild buffer conditions, and these two features even make this system a more convenient purification system to be utilized in various low background-requiring applications, such as mapping of a protein of interest with its cellular targets (interactome). We recently applied this system to determine the JCV agnoprotein interactome successfully. Due to its aforementioned attractive features, we also utilized the “Twin Strep Tag II” and MagStrep affinity purification system to map the cellular targets of JCV LT-Ag and JCV Sm t-Ag, as described below (Figure 1A and Figure 2).

### 3.2. Analysis of JCV LT-Ag- and JCV Sm t-Ag-Binding Proteins by Silver Staining Prior to Mass Spectrometry Analysis

Three expression plasmids, as indicated in Figure 1B, were transfected into the HEK293T cells separately by the calcium-phosphate transfection method. We chose HEK cells for our transient expression experiments since HEK cells possess various glial-specific genes [76], which make them an ideal cell line for expressing neurotropic viral proteins such as JCV LT-Ag and JCV Sm t-Ag for proteomics studies (Figure 1A). Whole-cell extracts prepared from both control and experimental transfectants were separately incubated with “MagStrep Type 3 XT” magnetic beads to capture tagged LT-Ag and Sm t-Ag and their associated proteins. Then, the magnetic bead-bound proteins were washed, eluted with biotin, and resolved on a NUPAGE 4–12% Bis-Tris gradient gel followed by silver staining (Figure 1B). The T7-2xStrep peptide (control lane) is visible on the silver staining gel system (Figure 1B, lane 2). The comparison of the control lane with those of Sm t-Ag and LT-Ag (Figure 1B, lanes 3 and 4) shows a clear difference between them. Silver stain detected both Sm t-Ag and LT-Ag on the gel, indicated by arrows. Two additional strong protein bands on the LT-Ag lane (lane 4) were also observed, which most likely resulted from translation of the alternatively spliced transcripts of the JCV LT-Ag, as previously reported [74,75]. A large amount of cellular host proteins that interact with either Sm t-Ag (lane 3) or LT-Ag (lane 4) is clearly visible when compared to the control lane (lane 2).

**Figure 3:**
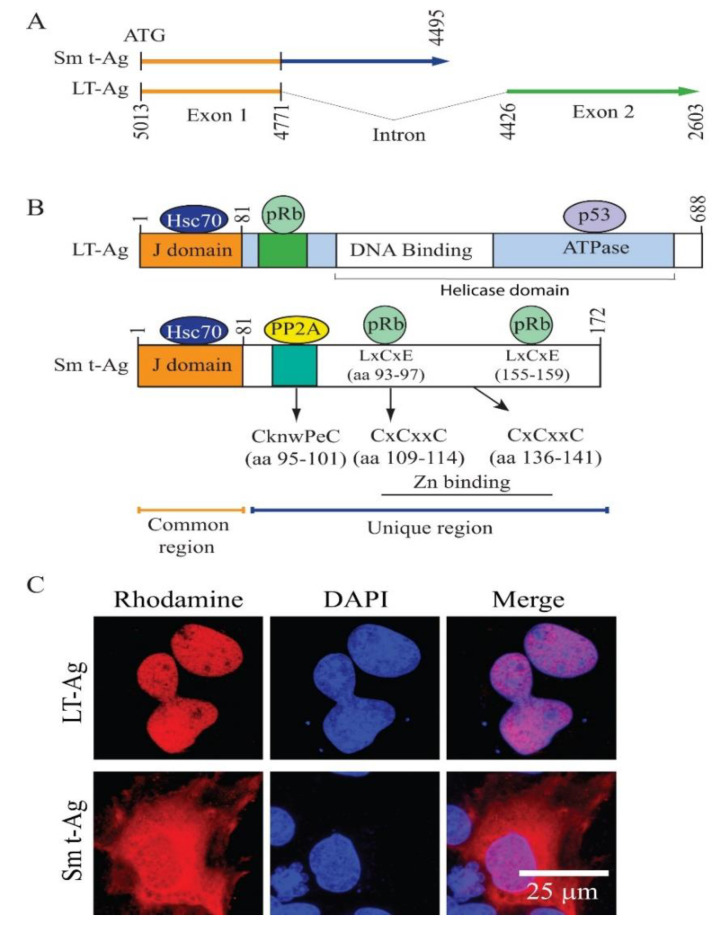
(**A**) Schematic representation of JCV Mad-1 early coding region. The common coding region between JCV LT-Ag and Sm t-Ag constitutes the exon 1. The unique coding regions are also indicated for each protein. Numbering is according to JCV Mad-1 strain (GenBank # NC_001699, formerly J02226). (**B**) A schematic representation of various functional domains on both LT-Ag and Sm t-Ag is indicated. Selected host protein-binding regions are also indicated for both proteins. The positions of the two pRb binding motifs (LxCxE) [77], the two Zn-binding clusters (CxCxxC), and the PP2A binding motif (CknwPeC) [77] on Sm t-Ag unique region are also shown. (**C**) Subcellular distribution of JCV LT-Ag and Sm t-Ag are analyzed on SVG-A cells by immunocytochemistry (ICC). The pcDNA3.1(+)-T7-2xStrep-JCV Sm t-Ag or pcDNA3.1(+)-T7-2xStrep-JCV LT-Ag plasmids were separately transfected into SVG-A cells on glass-chamber slides and analyzed by ICC using α-T7 monoclonal antibody, as described [2].

In parallel, the eluted samples containing the Sm t-Ag- and LT-Ag-bound proteins were also analyzed by Western blotting using α-T7 antibody (Figure 1C, lanes 3 and 5, respectively). The protein bands related to JCV Sm t-Ag and LT-Ag, which are visible on the silver staining gel (Figure 1B, lanes 3 and 4), were also detected by Western blotting, confirming their identity. For mass spectroscopy analysis, the same set of samples were also partially resolved on a NUPAGE 4–12% Bis-Tris gradient gel to remove small contaminants such as “avidin” during the elution process prior to tryptic digestion (Figure 1D). As shown in Figure 1D, the gel areas encased by the dash-lined rectangles were excised from the gel, digested with trypsin, and processed for proteomics analysis. A flow chart provided in Figure 2 summarizes each step in the proteomics studies designed to identify the JCV LT-Ag- and Sm t-Ag-associated proteins in cells by mass spectroscopy. In addition, the splicing patterns of the JCV LT-Ag and Sm t-Ag, as well as the cellular distribution of both proteins, are shown in Figure 3A–C.

### 3.3. Data Enrichment and Analysis

The AP/MS data obtained from two independent affinity purifications were further subjected to an enrichment process based on several data exclusion criteria, as follows: (1) Interaction between JCV LT-Ag and its targets or between JCV Sm t-Ag and its targets had to be present in both AP/MS data runs with more than two significant peptides. (2) No background was allowed in data enrichment except for a few cases where the significant peptide numbers were high in that the background showed only one significant peptide.

Two independently run AP/MS data sets for JCV LT-Ag- and Sm t-Ag-interacting proteins yielded 622 total targets (Figure S1) and, after eliminating backgrounds based on the criteria stated above, the new selected total was 139 hits, 68 for LT-Ag and 71 for Sm t-Ag (Figure S2). Among these interactions, 27 targets were common to both proteins (Figure S3). Our data also revealed 41 unique cellular interactions for JCV LT-Ag with at least two significant peptides, including three cases with low background (one peptide) (Figure S4) and 41 unique interactions for JCV Sm t-Ag with at least two significant peptides, including in three cases with low background (one peptide) (Figure S5). 

With respect to the detection of the high number of the specific peptides for a particular protein in the AP/MS data, our proteomics data showed that the LT-Ag and Sm t-Ag target several specific host proteins. For instance, JCV LT-Ag strongly interacts with FBWX11, evidenced by the highest number of significant peptides detected in the first and second runs (17 and 18 peptides, respectively). FBWX11 is an F box protein and a major component of the E3 ubiquitin ligase complex. In the first AP/MS run, this hit had no background. In the second run, however, the gel control had four significant background peptides. We think that it is due to the cross-contamination during the gel run. JCV LT-Ag also showed a strong interaction with protein phosphatase 4 (PP4) subunits (SMEK1 (PPP4R3A), PPP4R2, and PPP4C) and vacuolar v-ATPase, which is evident from the detection of a large number of peptides belonging to these proteins in the proteomics data (Figure S1). Additionally, this proteomics data confirms the interaction of JCV LT-Ag with the human replication protein A (RPA1), which was previously shown to interact with the SV40 LT-Ag [78]. In JCV Sm t-Ag proteomics data, AIFM1, an apoptosis-inducing factor, exhibited with the highest significant peptides (25 and 27 in first and second runs, respectively) with no background. The data also showed a strong interaction of JCV Sm t-Ag with both a chromatin-remodeling protein, Smarca 5, and the SDH subunits A and B (Figure S1). Our AP/MS data also confirmed the previously reported JCV Sm t-Ag interaction with protein phosphatase 2A (PP2A) with high numbers of the significant peptides (Figure S1) (Table 1) [72,77]. 

We were aware of the fact that some of these interactions may have resulted from the viral protein overexpression or nonspecific interactions of the host proteins with these tumor antigens [79]. Therefore, the AP/MS data were analyzed with greater scrutiny. The nonspecific-interacting partners of these tumor antigen proteins or contaminants may include translation initiation and elongation factors, ribosomal proteins, heat shock proteins, desmin, peripherin, keratin, vimentin, myosin, cofilin, ribonucleoproteins, tubulin, actin, and others, as described by Engeland et al. [79]. We discarded most of these possible artifacts and contaminating proteins, which showed interaction with LT-Ag and Sm t-Ag but exhibited high background. However, some of these proteins may still be genuine binding partners to LT-Ag and Sm t-Ag antigens, such as heat shock proteins and myosin and actin network proteins, which exhibited strong interaction with tumor antigens with zero background. Of note, several host proteins that appeared in our AP/MS data were previously reported to interact with LT-Ag and Sm t-Ag by different methods. Some of these interactions are now validated by the current AP/MS data (Table 1 and Table 2).

### 3.4. Building the Interactomic Maps for JCV LT-Ag- and JCV Sm t-Ag-Interacting Proteins Using STRING Database Web Tool

**Figure 4:**
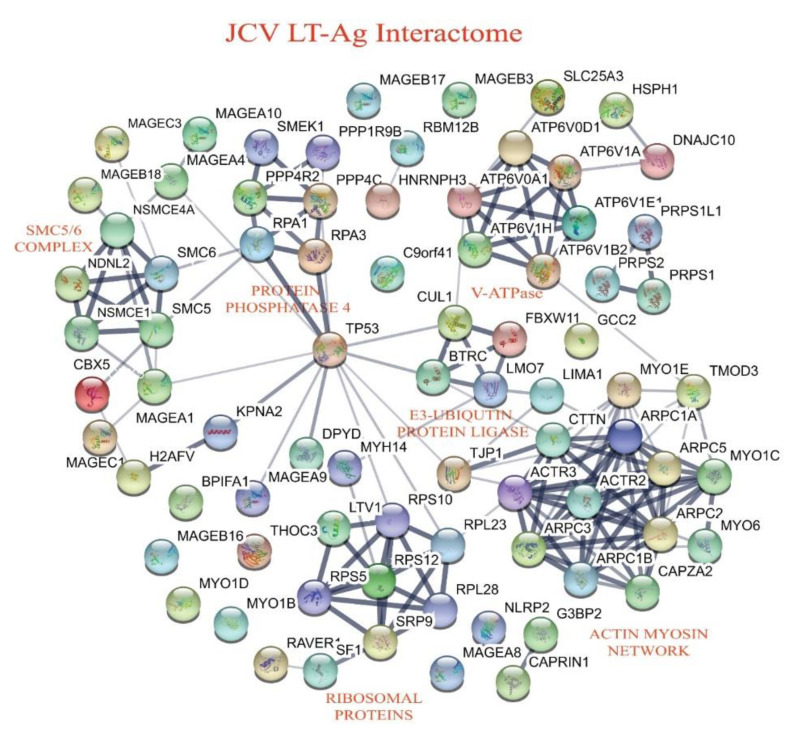
Building an interactome map for the host proteins targeted by JCV LT-Ag using “STRING database”. Analysis of the JCV LT-Ag interactome using the STRING database showed that JCV LT-Ag targets various protein complexes and networks including V-ATPase, Smc5/6 complex, PP4–PP1 complex, E3-Ubiquitin-protein ligase, ribosomal proteins, actin-myosin network, and others (Table 3).

**Figure 5:**
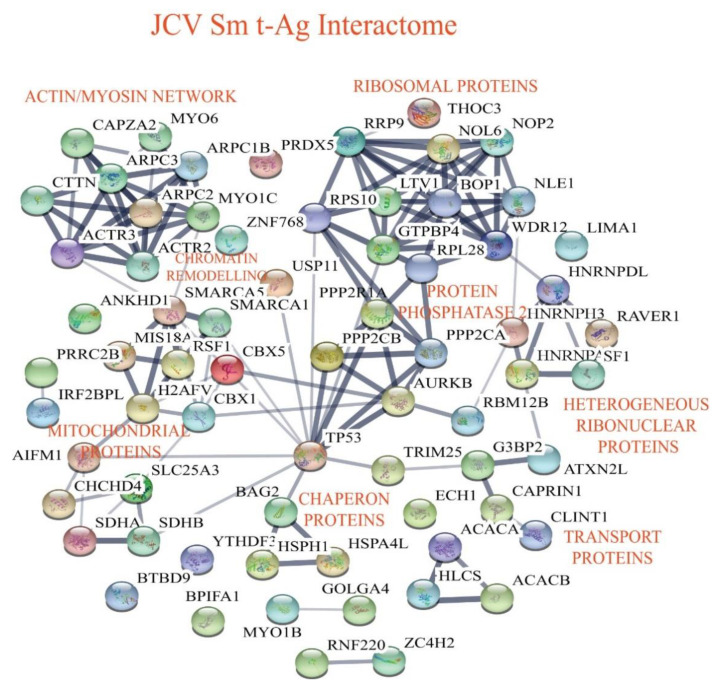
Analysis of the JCV Sm t-Ag interactome using “STRING database”. JCV Sm t-Ag primarily targets chromatin-remodeling proteins, mitochondrial proteins, PP2A complex proteins, chaperone proteins, Zn-binding proteins, heterogeneous ribonuclear proteins, ribosomal proteins, actin-myosin network, and others (Table 4).

**Figure 6:**
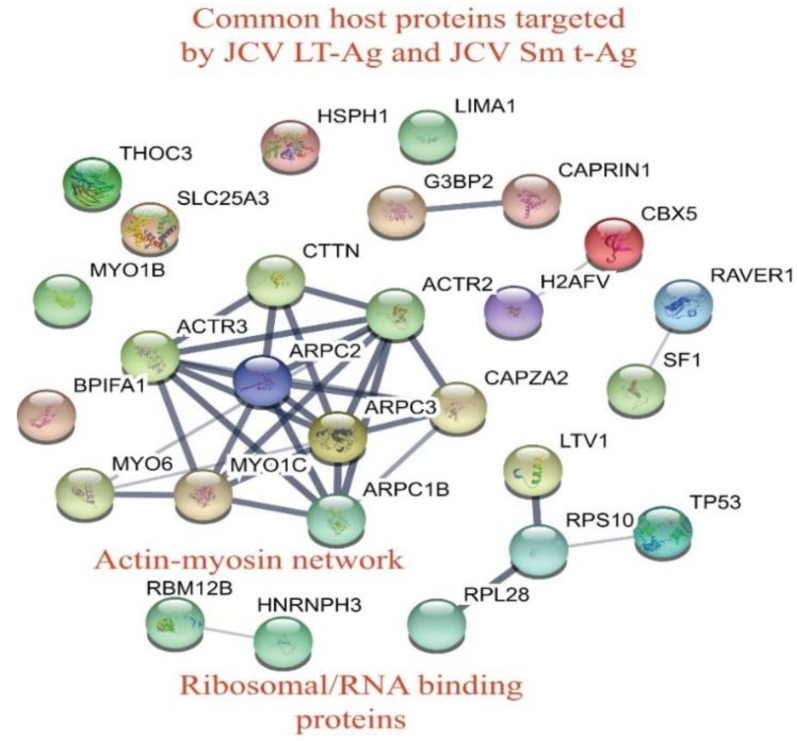
Analysis of the common host tumor antigen interacting proteins using “STRING database”. Both JCV LT-Ag and Sm t-Ag also target common host proteins including actin-myosin network, ribosomal/RNA binding proteins, and others (Table 5).

The “STRING database”—(Search Tool for the Retrieval of Interacting Genes/Proteins) is a comprehensive protein–protein interaction network-building web resource (https://string-db.org) and is a very useful tool to analyze the proteomics data. We used this database tool to build JCV LT-Ag (Figure 4) and JCV Sm t-Ag (Figure 5) interactomes. As mentioned above, after extensive analysis of the proteomics data, we realized that both tumor antigens also target common host proteins (Figure 6), perhaps due to the fact that both LT-Ag and Sm t-Ag share sequence homology in their N-terminus (Figure 3A,B). As shown in Figure 4 and Table 3, JCV LT-Ag primarily targets the following protein complexes, v-ATPase complex, Smc6 complex, protein phosphatase 1/4 complex, E3-ubiquitin protein ligase, and networks, ribosomal protein network and actin-myosin network, and other individual proteins. Interestingly, some of these LT-Ag targets possess essential enzymatic activities.

On the other hand, JCV Sm t-Ag interacts with the host proteins and protein complexes involved in critical cellular functions such chromatin-remodeling proteins, dephosphorylation (PP2A complexes), chaperoning, and cellular transport. JCV Sm t-Ag also binds to Zn-binding proteins, heterogeneous ribonucleoproteins, and organelle/protein networks, including ribosomal protein, actin-myosin network, mitochondrial proteins, and a number of other cellular proteins listed in Table 4 and Table 5. Note that Sm t-Ag exhibits both nuclear and cytoplasmic distribution patterns in cells (Figure 3C). This feature of Sm t-Ag is reflected through its interaction with both the nuclear and cytoplasmic proteins (Figure 5). It is surprising that we also observed Sm t-Ag interaction with p53, an important tumor suppressor protein (Figure 5 and Table 4), which is usually targeted by the polyomavirus LT-Ag and by the various other viral oncogenic proteins. This proteomics data also shows that both JCV LT-Ag and JCV Sm t-Ag interact with common host proteins that are part of the actin-myosin network and ribosomal/RNA binding proteins and other individual proteins (Figure 6, Table 5). 

#### 3.4.1. JCV LT-Ag Targets Smc5/6 Complex

As shown in Figure 4 and Table 3, JCV LT-Ag targets several important protein assemblies in host cells including Smc5/6 complex, v-ATPases, E3-ubiquitin protein ligases, and others. We will elaborate on these new cellular targets as possible new pathways/mechanisms employed by JCV LT-Ag to induce its oncogenic process. The SMC (structural maintenance of the chromosomes) protein complex plays essential roles in maintaining the genomic integrity, repairing the damaged DNA, and chromosome segregation. The core of these groups of proteins is highly conserved and dependent on the combination of the formation of the core heterodimeric structures. They are grouped into the four trimeric Smc-kleisin complexes [94] as follows: (1) The condensin I and II complexes composed of Smc2/4 heterodimeric and non-Smc (kleisin) components play roles in regulation of the mitotic chromosome condensation [95,96]. (2) The cohesin complex composed of Smc1/3 heterodimers and kleisin components plays roles in holding sister chromatids together until they are segregated during the anaphase [97,98]. (3) The Smc5/6 complex with kleisin elements is best known for its involvement in DNA repair and maintaining genomic stability [99,100,101]. (4) The last group is named “the dose compensation complex” and plays roles in heterochromatin formation and gene silencing [102]. Structurally, the core of the Smc complex resembles a V-like shape and is composed of heterodimers. Each arm alone represents a monomer on the V-like shape. There is a hinge region located toward the central portion of each monomer, providing flexibility to the two flanking regions for folding back onto each other to form one of the arms on the V-like shape. The heterodimers interact with each other through the hinge region of each protein at the bottom of the converging arms. As mentioned above, the third member in the trimeric Smc complex, named kleisins (Nse 1–6), bridges the opening end of the V-like shape to complete the ring structure [103]. However, the Nse2 does not participate in the bridging process but interacts with Smc5 toward the middle portion of the protein. The head portion of each arm on the V-shape structure forms a globular nucleotide-binding domain for ATP binding and hydrolysis. Structurally, the heterodimeric proteins (Smc 1–6) provide a scaffolding function to the complex, whereas the non-Smc proteins (kleisins) were found to have various enzymatic functions, such as sumoylation [104] and E3-ubiquitination, suggesting that kleisins coordinate the substrate selection and modification during chromosome segregation and DNA repair [105].

A couple of viral proteins were also reported to target Smc5/6 complex in a direct or indirect manner. For example, the Hepatitis B X protein hijacks the DNA damage binding protein 1 (DDD1)-containing E3 ligases to target the Smc5/6 complex for degradation [106]. The papillomavirus E2 protein is another example of a viral protein to target Smc5/6 complex, which was reported to play a role in the maintenance of the viral genome [107].

Our current proteomics data also revealed the targeting of Smc5/6 complex by LT-Ag in both data runs (Figure 4, Table 3). JCV-LT Ag-interacting proteins include not only the scaffolding proteins of the Smc5/6 complex but also those with enzymatic activities ((NSMCE1 (Nse1), a E3-ubiquition ligase) and other kleisin members, including NSMCE4A (Nse4), NDSL2 (Nse3), and melanoma-associated proteins (MAGEC3, MAGEB18, MAGEA1, MAGEC1). Some of these MAGE subunits have binding activity to Smc5/6 complex. The significance of the JCV LT-Ag interaction with Smc5/6 complex is not completely understood with respect to both the viral life cycle and cell transformation. We speculate that LT-Ag most likely affects the ATPase, sumoylation or E3-ligase activity Smc complex members. Whether such modifications on Smc complex members contribute to the genomic instability or impairment in the Smc5/6 complex-mediated DNA repair, impairment in the resistance to the genotoxic and UV-mediated insults. Such possibilities need to be further investigated with respect to oncogenicity by LT-Ag.

**Figure 7:**
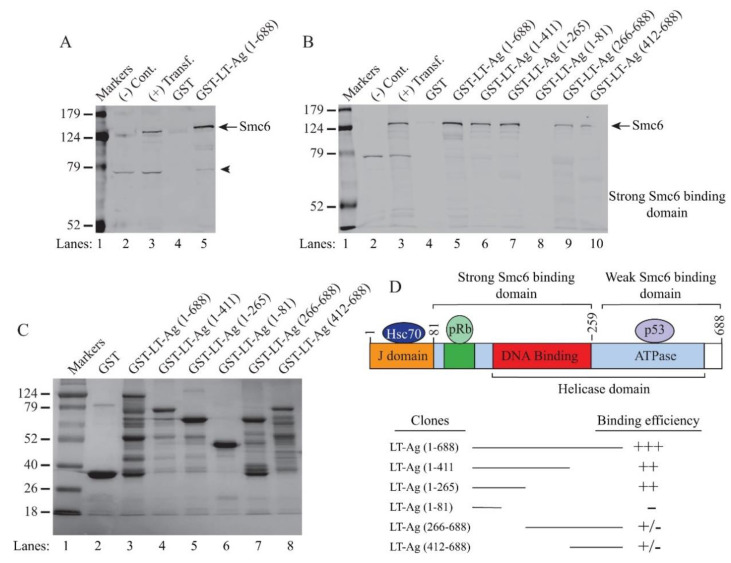
Validation of JCV LT-Ag interaction with Smc6. (**A**) Analysis of the interaction of JCV LT-Ag with Smc6 by GST pull-down assays, as described in the Materials and Methods. Briefly, whole-cell extracts (0.5 mg) prepared from HEK293T cells transfected with the FLAG-tagged-Smc6 expression plasmids (lanes 3 and 5) were incubated with either GST (2 µg) alone (lane 4) or GST-JCV LT-Ag (2 µg) (lane 5). After washing, proteins interacting with GST or GST-JCV LT-Ag were analyzed by Western blotting using an α-flag antibody for detecting FLAG-tagged Smc6. (**B**) Mapping the interaction domain(s) of JCV LT-Ag with Smc6 by GST pull-down assays. In parallel to the experiments described for panel A, similar GST pull-down experiments were also carried out for mapping assays, as described in Materials and Methods. Large T antigen-binding proteins were analyzed by Western blotting using an α-flag antibody for detection of FLAG-tagged Smc6. In lanes 2 and 3 (A and B), whole-cell extracts from untransfected [(−) Cont.] and transfected cells [(+) transf.] were loaded as negative and positive controls, respectively. (**C**) Analysis of GST, GST-JCV LT-Ag, and GST-JCV LT-Ag mutant proteins by SDS-12% PAGE. GST and GST-JCV LT-Ag and GST-JCV LT-Ag mutants were produced in bacteria and affinity purified, as previously described [73]. Four-microgram aliquots of each protein were resolved on a SDS-12% PAGE and stained by coomassie blue. (**D**) A graphical presentation of LT-Ag domain and the binding efficiency of LT-Ag to Smc6. Binding efficiencies were scaled as +++: Strong, ++: Moderate, and +/−: Weak binding.

We have further validated the interaction of LT-Ag with Smc6 from the Smc5/6 complex by protein–protein interaction (GST pull-down assays) and immunocytochemistry studies. To achieve this, whole-cell extracts prepared from HEK293T cells expressing a FLAG-tagged Smc6 (Figure 7A, lane 3) or FLAG tag alone were incubated with GST or GST–JCV LT-Ag fusion (Figure 7A) proteins immobilized on glutathione–sepharose beads followed by an analysis by SDS-PAGE/Western using an α-FLAG antibody, as described in Materials and Methods. As shown in Figure 7A, GST pull-down experiments resulted in a strong interaction between JCV LT-Ag and Smc6 (Figure 7A, lane 5) while GST control showed no binding (lane 4), indicating a specific interaction between JCV LT-Ag and Smc6. Additionally, we performed a series of GST pull-down assays using JCV LT-Ag truncation mutants in order to map the interaction domains of JCV LT-Ag with Smc6 (Figure 7B). The J domain of JCV LT-Ag (aa 1–81) did not show any interaction with Smc6 (Figure 7B, lane 8) but the regions encompassing amino acids 1–265 and 1–411 manifested strong interactions (lanes 6 and 7).

The C-terminal region of JCV LT Ag (aa 266-688) (lanes 9 and 10) also showed a weak interaction, suggesting that JCV LT-Ag might contain two SMC binding domains required for the interaction with Smc6: A strong interaction domain encompassing aa 82–266 at the N-terminus and a weak interaction domain encompassing aa 412–688 at the C-terminus. The SDS-PAGE analysis of the full-length GST-JCV LT-Ag and that of the deletion mutants, where truncated forms of JCV LT-Ag fused to GST, is shown in Figure 7C. The strengths of JCV LT-Ag domain interactions with Smc6 based on the GST pull-down assays are depicted graphically in Figure 7D. 

**Figure 8:**
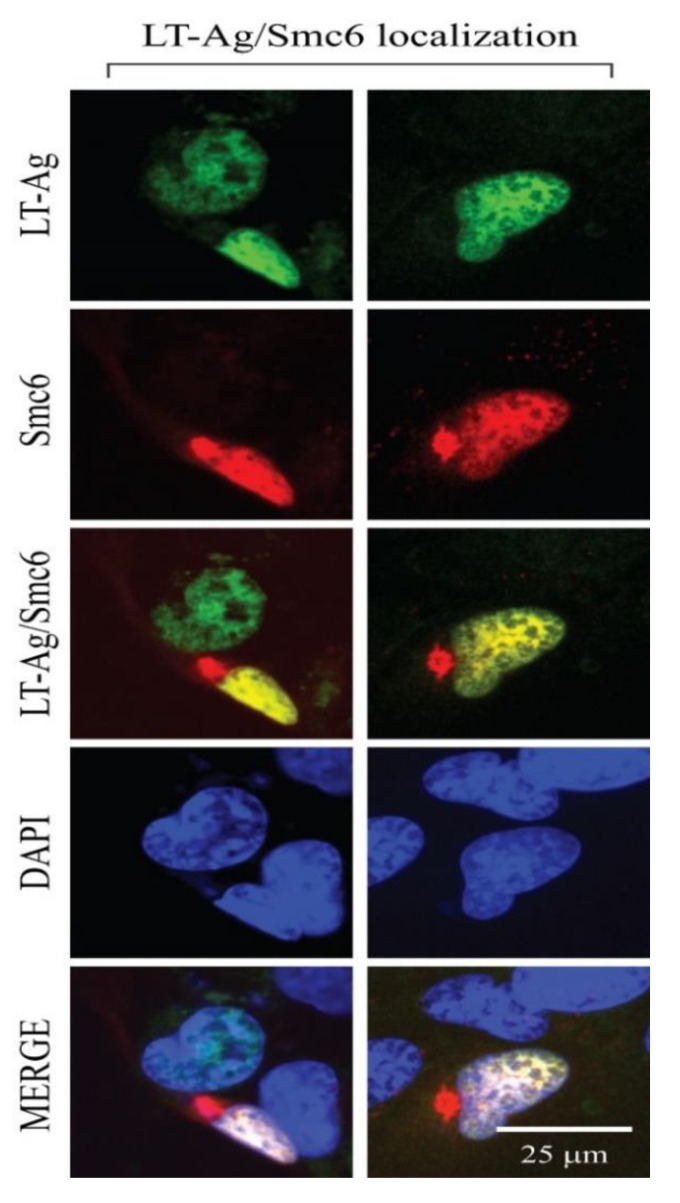
Analysis of the subcellular distribution of JCV LT-Ag and Smc6 by immunocytochemistry (ICC). T7-tagged LT-Ag and FLAG-tagged Smc6 expression plasmids were co-transfected into SVG-A cells and the subcellular localization of both proteins were analyzed by ICC, as described in the Materials and Methods. Briefly, at 16 h post-transfection, cells were transferred to glass-slide chambers and incubated for an additional 24 h. Cells were then fixed in cold acetone and incubated with a combination of α-T7 polyclonal and α-FLAG monoclonal primary antibodies overnight. After extensive washing with 1× PBS, the slide chambers were incubated with the appropriate FITC- or rhodamine-conjugated secondary antibodies, and microscopic images were obtained under a fluorescence microscope, as described in Materials and Methods. Scale bar: 25 µm.

In parallel, we also performed immunocytochemistry (ICC) to support our findings from the GST pull-down experiments. For this purpose, SVG-A cells were co-transfected by both a JCV LT-Ag and a flag-tagged Smc6 expression plasmids, and ICC experiments were performed, as described in Materials and Methods. As shown in Figure 8, both JCV LT-Ag and Smc6 showed a nuclear and overlapping localization, which is consistent with our findings from the protein–protein interaction assays (GST pull-down, Figure 7). Additionally, the proteins that were previously shown to interact with JCV LT-Ag are tabulated on Table 2. Some of those proteins were revalidated by our current proteomics data, including p53, Yb-1, F-box protein (BTrCP1/2), etc. Interestingly, our proteomics data did not detect an interaction between LT-Ag and pRb under our experimental conditions. It is likely that such an interaction was not stable in our experimental conditions.

#### 3.4.2. JCV LT-Ag Targets Vacuolar (V)-ATPases

V-ATPases are one of the largest multi-subunit (14 subunits) protein complexes located on the membranes of the intracellular organelles such as endosomes and lysosomes [108], although some cells have them on their plasma membranes, as well, to carry out the cell-specific functions such as renal acidification, bone resorption, sperm maturation, and hemostasis of the cytoplasmic pH. V-ATPases are composed of two main domains: (1) An ATP hydrolytic domain located on the surface of the organelle membrane and (2) the proton-translocating domain located within the membrane itself. Their function is tightly regulated by the reversible dissociation of the V_1_ and V_o_ domains [109]. The primary function of theses complexes is to acidify the lumen of the organelle by the proton pumping inward into the organelle. Such an acidification process is required for the uncoupling and sorting of the internalized receptor-ligand complexes and lysosomal degradation of the substances [110]. Thus, the acidification process of such organelles is crucial for many biological processes, including protein degradation, membrane trafficking, and transport of the small molecules [109].

V-ATPases play critical roles during some viral infections as well. For example, the internalization of the Semlike Forest virus (SFV) depends largely on the activity of V-ATPases, evidenced by the insufficient amount of viral particles produced when the cells are treated with a V-ATPase inhibitor, Bafilomycin [111]. Another example is that the HIV-1 Nef interacts with the V1-H subunit of ATPase and decreases the expression of CD4 on the surface of the infected cells and thereby connects the Nef to endocytic machinery in modulation of the viral pathogenesis [112]. The importance of the acidification of the vacuoles by V-ATPases in human cytomegalovirus (HCMV) maturation was also recently reported, showing that V-ATPase activity plays a critical role in the formation of the virion assembly compartments in the infected cells and, in the absence of which, there is a profound reduction in the production of infectious particles [113].

More importantly, our proteomics data also showed consistent results from each AP/MS run, where LT-Ag was also found to interact with both V1 and Vo domains of V-ATPAse. More specifically, the V1 subunits, E1, A, B2-brain isoform, and H, and “a” and “d” subunits of the Vo domains of V-ATPase appear to be targeted by LT-Ag (Figure 4 and Table 3). Currently, we do not know the significance of such a targeting with respect to the role of V-ATPase in JCV infection as well as in LT-Ag-mediated cell transformation. Both topics await further investigation.

#### 3.4.3. Targeting of E3-Ubiquitin Complex by JCV LT-Ag

Ubiquitination is a post-translational modification of proteins, carried out by the sequential activity of a group of ligases, including ubiquitin-activating (E1), ubiquitin-conjugating (E2), and ubiquitin-ligating (E3) enzymes, on the specific lysine residues of the target substrates [114]. The human genome encodes two E1, ~35 E2, and >700 E3 ubiquitin ligases [115] and ubiquitination is primarily known for the degradation of protein substrates upon the modification. However, in recent years, this post-translational modification has emerged as a critical mechanism by which it appears to regulate diverse biological processes, including various aspects of immune function and signaling pathways [116,117]. The E3-ubiquitin ligase complex is composed of both the core and the adaptor proteins. The Cullins make up the core, and Skp1 and various F-box proteins constitute the adopter proteins. The F-box proteins are even categorized in the WD-40 domain-containing (Fbxws), leucine zipper domain-containing (Fbxls), and other variable domain-containing (Fbxos) [118] proteins.

The interaction of SV40 LT-Ag with the core component of E3-ubiqutin ligase, Cul7, was reported almost two decades ago [119]. The follow-up studies then revealed that this targeting by SV40 LT-Ag contributes to cell transformation [120] by inhibiting the enzymatic activity of E3-ubiquitin ligase complex [121]. In addition to SV40, the interaction of the MCPyV tumor antigens with the subcomponents of the E3-ubiquitin ligase complex was also investigated by Kwun et al. [122]. It was demonstrated that MCPyV Sm t-Ag, rather than LT-Ag, targets the SCF^Fbw7^ through its LT-Ag stabilization domain [122,123] and contributes to cell transformation by stabilizing the protein levels of the SCF^Fbw7^ cellular targets including c-Myc and cyclin E [122]. Our current proteomics study also identified the targeting of the E3-ubiquitin complex by JCV LT-Ag, which includes the Fbxw11, Cul1, LMO7, and BTRC from this family (Figure 3, Table 4). The proteomics data also revealed that, in contrast to SV40 LT-Ag targeting of the Cul7 core protein, JCV LT-Ag showed a differential interaction with the E3-ubiquitin ligase members, namely, with Cul1 and Fbxw11, in both of our data runs. The significance of this differential interaction is currently unknown and awaits further investigation with respect to cell transformation by JCV LT-Ag. 

Our data also showed an interaction of LT-Ag with “the protein phosphatase 4” group of proteins, actin/myosin network, and ribosomal proteins. The targeting of the phosphatases by several polyomavirus small t antigens, (JCV, BKV, SV40, and MCV) [46,72,77,124] were previously reported. However, this is the first time that we report an interaction of JCV LT-Ag with such a group of phosphatases.

#### 3.4.4. JCV Sm t-Ag Targets Chromosome-Remodeling Protein Complexes, Smarca1 and 5

In addition to LT-Ag interactome, we also built the JCV Sm t-Ag interactome using the STRING web tool using our proteomics data. JCV Sm t-Ag exhibited interactions with various critical protein complexes and networks, including chromatin-remodeling proteins (Smarca1 and Smarca6), mitochondrial proteins such as an apoptosis initiator protein 1 (AIFM1) and succinate dehydrogenase subunits A and B (SDHA/B), phosphatases, chaperon and transport proteins, ribosomal proteins, actin/myosin network proteins, and others (Figure 5, Figures S1 and S5, Table 4). Note that some of these interactions contain common targets both for LT-Ag and Sm t-Ag (Figure 6, Table 5).

Since polyomavirus Sm t-Ags cooperate with LT-Ag in transformation of cells in tissue culture and animal model systems [40,46,125,126,127,128], we reasoned that some of the new Sm t-Ag targets have an apparent relevance to the cell transformation pathways. As such, the chromatin-remodeling proteins (Smarca1 and 5) could be, for example, categorized into this category. Smarca1 and 5 proteins (SW1-SNF-related, matrix-associated, actin-dependent regulator of chromatin A 1 and 5) are classified within the family of SWItching (SWI) defective/Sucrose Non-Fermenting (SNF) gene family (SWI/SNF). The proteins in this family are known to play essential roles in chromatin remodeling, [129] and both Smarca1 and Smarca5 have intrinsic ATPase and helicase activities [129] to fulfill their remodeling activity. In addition to chromatin-remodeling activity, Smarca5, in particular, was shown to participate in DNA damage repair to resolve the lesion-stalled transcription [130]. Mouse knock-out studies revealed an embryonic lethal phenotype for Smarca5. Smcarca5 is also reported to associate with “extrachromosomal Ewing’s sarcoma” [131] and plays an essential role in the proliferation and differentiation of both hematopoietic stem and progenitor cells [132]. Thus, it is feasible that the targeting of Smarca1 and Smarca5 chromatin-remodeling proteins by Sm t-Ag may have implications with respect to the restrictions on the Smarca5-mediated gene expression and DNA repair and, thus, such a targeting may ultimately contribute to cell transformation.

#### 3.4.5. Interaction of JCV Sm t-Ag with Succinate Dehydrogenase (SDH) Subunits A and B

Succinate dehydrogenase (respiratory complex II) is a mitochondria inner membrane-bound enzyme, composed of four nuclear-encoded subunits, A, B, C, and D. Its primary function is to convert succinate to fumarate, while passing electrons to reduce ubiquinone. SDHA subunit carries FAD (flavin adenine dinucleotide cofactor) while SDHB subunit has three Fe-S clusters [133]. SDHA can act as a tumor suppressor and contributes to the formation of paraganglioma [134,135]. Since SDH is an essential housekeeping enzyme located in mitochondria, its involvement in tumorigenesis was not expected. It was surprising to discover that the inherited or somatic mutations in SDH genes could lead to different cancers such as paraganglioma or phaeochromocytoma catecholamine-producing neuroendocrine tumors [136,137,138,139], gastrointestinal tumors (GIST) [140]. A very recent work indicated that SDH mutations may lead to the defects in homologous DNA repair [141] and disruption in chromatin signaling [142]. Collectively, these studies implicated that SDH plays a role in tumor suppression as well as in chromatin modeling. Thus, the targeting of the SDHA and SDHB subunits by JCV Sm t-Ag may result in the inhibition of the catalytic activity of the enzyme and cause succinate accumulation. This SDH inhibition may the lead to defects in homologous DNA repair and chromatin remodeling and, thus, contribute to cell transformation. 

**Figure 9:**
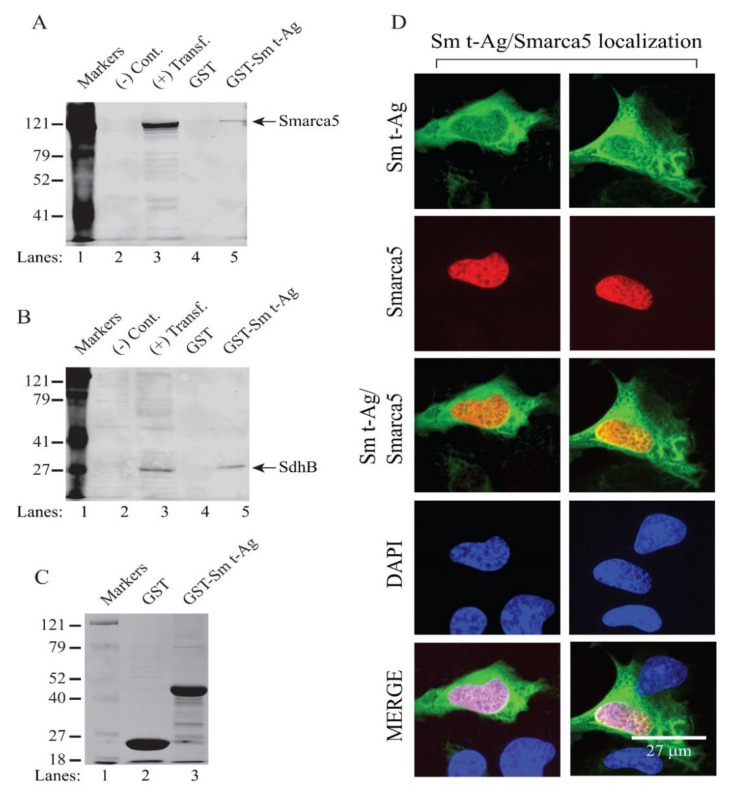
Validation of the JCV Sm t-Ag interaction with SDHB and Smarca5. (**A**,**B**) Analysis of the interaction of JCV Sm t-Ag with Smarca5 (**A**) and that of SDHB (**B**) by GST pull-down assays was carried out, as described in Materials and Methods. Whole-cell extracts (0.5 mg) prepared from HEK293T cells transfected with the FLAG-tagged Smarca5 expression plasmid (lanes 3 and 5) were incubated with either GST (2 µg) alone (lane 4) or GST-JCV LT-Ag (2 µg) (lane 5). After washing, proteins interacting with GST or GST-JCV Sm t-Ag were analyzed by Western blotting using an α-flag antibody for detection of flag-tagged Smarca5. (**B**) In parallel, similar GST pull-down experiments were carried out using whole-cell extracts prepared from HEK293T cells transfected with a FLAG-tagged-SDHB expression plasmid (lanes 4 and 5). In lanes 2 and 3 (A and B), whole-cell extracts from untransfected [(−) Cont.] and transfected cells [(+) transf.] were loaded as negative and positive controls, respectively. (**C**) Analysis of GST and GST-Sm t-Ag proteins by SDS-12% PAGE followed by coomassie blue staining, as described for Figure 8C. (**D**). Analysis of the subcellular distribution patterns of JCV Sm t-Ag and Smarca5 by ICC. SVG-A cells were co-transfected with both T7-tagged Sm t-Ag and FLAG-tagged Smarca5 expression plasmids and the subcellular localization of both proteins was analyzed by ICC, as described under Figure 8, using α-T7 polyclonal and α-FLAG monoclonal primary antibodies and FITC or rhodamine-conjugated secondary antibodies. Finally, microscopic images were obtained under a fluorescence microscope, as described under the Materials and Methods. Scale bar: 27 µm.

We also further validated the interaction of Sm t-Ag both with Smarca5 and SDHB proteins by protein–protein interaction studies. Some proteins that were previously shown to interact with Sm t-Ag are also tabulated on Table 1. Some of those proteins were also revalidated by our proteomics data, including PP2A, Hps70, etc. To further validate the interaction of Sm t-Ag with either Smarca5 or SDHB, we performed GST pull-down experiments using whole-cell extracts prepared from HEK293T cells expressing a flag-tagged Smarca5 (Figure 9A, lane 5) or flag-tagged SDHB (Figure 9B), as described in the Figure 9 legend. Both assays clearly demonstrated that JCV Sm t-Ag specifically interacts with both Smarca5 (Figure 9A) and SDHB (Figure 9B), which is evident from the fact that no visible interaction was observed with GST alone in both cases. Analysis of the GST-Sm t-Ag fusion protein by coomassie blue staining is shown in Figure 9C. In parallel to the GST pull-down assays, the cellular distribution patterns of Sm t-Ag along with Smarca5 (Figure 9D) were also analyzed by microscopy imaging assays. SVG-A cells were co-transfected with both a JCV Sm t-Ag and flag-tagged Smarca5 expression plasmids. The immunocytochemistry experiments were performed, as described in Materials and Methods. As shown in Figure 9D, as expected, Smarca5 shows an exclusive nuclear localization pattern, indicating an interaction with JCV Sm t-Ag in the nucleus.

### 3.5. Analysis of the LT-Ag and Sm t-Ag Proteomics Data Using FunRich Program

Proteomics data were also analyzed using the “FunRich program [66,67]. FunRich allowed us to classify AP/MS data based on the different categories such as biological processes, biological pathways, cellular components, molecular functions, protein domains, transcription factors, site of expressions, and clinical phenotypes. FunRich analysis revealed that JCV LT-Ag primarily targets the growth-promoting pathways (Figures S6A and S8A) and the proteins containing MYSc, IQ, Beta-TrCp-D and WD40 domains (Figures S6E and S8E). JCV Sm t-Ag, on the other hand, primarily interacts with the host proteins that play functional roles in cellular transport and recycling pathways (Figure S7C). For further FunRich analysis information, please refer to Figures S6–S8.

## 4. Conclusions

The early regulatory proteins of JCV tumor antigens, LT-Ag and Sm t-Ag, are produced by the alternative splicing of the viral early transcripts [13] (Figure 3A). Both play critical roles in the viral life cycle [48,72,92,143,144,145]. LT-Ag, in particular, plays an essential role in viral DNA replication, where it binds to the viral origin as a double hexamer manner, unwinds it, and initiates the DNA replication in both directions [143,144,145]. In the meantime, it transactivates the viral late promoter while inhibiting the early promoter by an auto-regulatory loop mechanism [146]. The regulatory role of the Sm t-Ag in viral DNA replication and transcription is less clear, other than the fact that it promotes various growth-promoting pathways [48,72]. The oncogenic potential of both proteins was previously tested both in tissue culture [54,147] and experimental animals [57,58,60,61,148,149]. Since JCV genome was detected in various human tumors [150,151,152,153], it is suggested that the oncogenic tumor antigens of JCV could also play a role in the induction of some of the human tumors, as seen by those of MCPyV [16,51,154]. In an attempt to further unravel their cellular targets and thereby open new avenues to explore their oncogenic mechanisms, in this work, we employed a proteomics approach to identify the partners of both proteins in cells. Through the enrichment of the two independent AP/MS runs, we identified 139 total proteins, which potentially interact with both JCV LT-Ag and JCV Sm t-Ag, after eliminating backgrounds based on the criteria stated in the text. Target wise, in contrast to JCV Sm t-Ag, it is clear that JCV LT-Ag primarily targets the protein complexes with various essential cellular enzymatic activities such as ATPase, phosphatase, and ubiquitin ligase while Sm t-Ag mostly associates with those having phosphatase, chaperone and chromatin-remodeling functions,. Lastly, this proteomics study presents itself as a comprehensive data source for the scientific community in the viral oncology field (particularly for those who are in the polyomavirus field) to open up new avenues for the characterization of those interactions in order to gain further insights into the new pathways in tumor progression pathways, mediated by these viral oncoproteins, which could further pave the way for the identification of new antitumor targets. 

## Figures and Tables

**Table 1:** Currently and previously reported host proteins that interact with JCV Sm t-Ag.

Proteins	Function	Reference
PP2A	Serine/Threonine phosphatase	[72,77]
Rb	Cell cycle regulation	[77]
Agnoprotein	JC virus gene regulation and replication	[72]
Hsp70	Chaperone	[80,81]
Smarca5	Helicase and ATPase activity	Current study
SDHB	Electron transport, oncogenesis	Current study

**Table 2:** Currently and previously reported host proteins that interact with JCV LT-Ag.

Proteins	Function	Reference
p53	Cell cycle regulation	[25,26,82,83]
pRb	Cell cycle regulation	[24,84]
BAG3	Inhibits Hsc70 ATPase activity	[85]
Beta-catenin	Contact inhibition	[86]
F-box protein (BTrCP1/2)	Ubiquitin protein ligase	[87]
CEBP	CCAAT DNA binding	[88]
Hsp70	Chaperone activity	[80,81]
IRS-1	Mediates insulin signaling	[89]
NF2	Membrane stabilizing protein	[90]
Pur alpha	Single strand DNA binding protein	[91]
Yb-1	Y-box binding protein	[71]
Agnoprotein	JC virus gene regulation, replication	[92]
Tst-1/Oct-6/SCIP	DNA binding, cell differentiation	[93]
Smc6	Structural maintenance of chromosomes	Current study

**Table 3:** Host proteins that interact with JCV LT-Ag.

Gene Code and Categories	Gene Name
***V-ATPAse***	
ATPV1E1	ATPase, H+ transporting, lysosomal 31 kDa, V1 subunit E isoform
ATP6V1A	V-type proton ATPase catalytic subunit A
ATP6V0D1	V-type proton ATPase subunit d 1 (Fragment)
ATP6V1B2	V-type proton ATPase subunit B, brain isoform
ATP6V0A1	V-type proton ATPase subunit a
ATP6V1H	ATPase, H+ transporting, lysosomal 50/57 kDa, V1 subunit H, isoform
***SMC5-SMC6 Complex***	
SMC5	Structural maintenance of chromosomes protein 5
SMC6	Structural maintenance of chromosomes protein 6
NSMCE1	Non-structural maintenance of chromosomes element 1 homolog
NSMCE4A	Non-structural maintenance of chromosomes element 4 homolog A
NDNL2	Non-structural maintenance of chromosomes element 3 homolog
MAGEA1, MAGEB18, MAGEC1, MAGEC3	Melanoma-associated antigens
***PPP4-PPP1 Complex***	
PPP4C	Serine/threonine-protein phosphatase 4 catalytic subunit
PPP4R2	Serine/threonine-protein phosphatase 4 regulatory subunit 2
SMEK1 (PPP4R3A)	Serine/threonine-protein phosphatase 4 regulatory subunit 3A
PPP1R9B	Neurabin-2
RPA1	Replication protein A 70 kDa DNA-binding subunit
RPA3	Replication protein A 14 kDa subunit
***E3-Ubiquitin Protein Ligase***	
FBXW11	Isoform B of F-box/WD repeat-containing protein 11
CUL1	cDNA FLJ58509, highly similar to Cullin-1
LMO7	LIM domain only protein 7
BTRC	Beta-transducin repeat containing isoform 4
***Ribosomal Proteins***	
RAVER1	Ribonucleoprotein PTB-binding 1
SF1	Splicing factor 1, isoform CRA_d
SRP9	Signal recognition particle 9 kDa protein
RPL23	Similar to ribosomal protein L23 (Fragment)
RPL28	60S ribosomal protein L28
RPS5	40S ribosomal protein S5 (Fragment)
RPS10	40S ribosomal protein S10
RPS12	40S ribosomal protein S12
LTV1	Protein LTV1 homolog
RBM12B	RNA-binding protein 12B
***Actin-Myosin Network***	
ARPC1A	Actin related protein 2/3 complex subunit 1A variant
ARPC1B	Actin related protein 2/3 complex, subunit 1B, 41 kDa
ARPC2	Arp2/3 complex 34 kDa subunit
ARPC3	Actin-related protein 2/3 complex subunit 3
ARPC5	Actin-related protein 2/3 complex subunit 5
MYO1C	Unconventional myosin-Ic
MYO1D	Unconventional myosin-Id
MYO1E	MYO1E variant protein, Unconventional myosin-Ie
MYO6	Unconventional myosin-VI
ACTR2	Actin-related protein 2
ACTR3	ARP3 actin-related protein 3 homolog (Yeast)
CTTN	Cortactin isoform a variant (Fragment)
CAPZA2	Capping protein (Actin filament) muscle Z-line, alpha 2 variant
***Others***	
DNAJC10	DnaJ homolog subfamily C member 1
C9orf41 (CARNMT1)	Carnosine N-methyltransferase
SRP9	Signal recognition particle 9 kDa protein
TMOD3	Tropomodulin-3
MAGEA4	Melanoma antigen family A, 4, isoform CRA_a
NLRP2	NACHT, LRR and PYD domains-containing protein 2
GCC2	RIP and coiled-coil domain-containing protein 2
DPYD	Dihydropyrimidine dehydrogenase [NADP (+)]
MYH14	Myosin-14
KPNA2	Importin subunit alpha
TJP1	Tight junction protein ZO-1
THOC3	THO complex subunit 3
BPIFA1	Isoform 2 of BPI fold-containing family A member 1
SLC25A3	Phosphate carrier protein, mitochondrial
G3BP2	Ras-GTPase activating protein SH3 domain-binding protein 2
HSPH1	Similar to heat-shock protein 105 kDa
TP53	Tumor protein p53
LIMA1	Epithelial protein lost in neoplasm beta variant
LTV1	Protein LTV1 homolog
CAPRIN1	Isoform 2 of Caprin-1
CBX5	Chromobox homolog 5 (HP1 alpha homolog, Drosophila)
H2AFV	Histone H2A

**Table 4:** Host proteins that interact with JCV Sm t-Ag.

Gene Code and Categories	Gene Name
***Chromatin remodeling***	
RSF1	Remodeling and spacing factor 1
H2AFV	Histone H2A.V
MIS18A	Protein Mis18-alpha
SMARCA5	FLJ79343, highly similar to SWI/SNF-related matrix-associated actin-dependent regulator of chromatin subfamily A member 5
***Mitochondrial proteins***	
CHCHD4	Coiled-Coil-Helix-Coiled-Coil-Helix Domain Containing 4
AIFM1	Apoptosis-inducing factor 1
ATAD3A	ATPase family AAA domain-containing protein 3A
SDHA	Succinate Dehydrogenase Complex Flavoprotein Subunit A
SDHB	Succinate Dehydrogenase Complex Iron Sulfur Subunit B
SLC25A3	Phosphate carrier protein, mitochondrial
PRDX5	Isoform cytoplasmic + peroxisomal of peroxiredoxin-5, mitochondrial
ECH1	Delta (3,5)-delta (2,4)-dienoyl-CoA isomerase, mitochondrial
**PP2A**	
PPP2R1A	Protein phosphatase 2 (formerly 2A), regulatory subunit A (PR 65)
PPP2CA	Serine/threonine-protein phosphatase 2A catalytic subunit alpha isoform
PPP2C2B	Serine/threonine-protein phosphatase 2A catalytic subunit beta isoform
***Chaperone proteins***	
HSPH1	cDNA FLJ51707, highly similar to heat-shock protein 105 kDa
BAG2	BAG family molecular chaperone regulator 2
HSPA4L	Heat shock 70 kDa protein 4L
***Transport proteins***	
CLINT1	Clathrin interactor 1 isoform 1
CAPRIN1	Isoform 2 of Caprin-1
***Zinc Binding proteins***	
ZNF768	cDNA FLJ59521, moderately similar to Zinc finger and SCAN domain-containing protein 2
ZC4H2	Isoform 3 of Zinc finger C4H2 domain-containing protein
***Heterogeneous ribonucleoproteins***	
HNRNPDL	cDNA FLJ60148, highly similar to Homo sapiens heterogeneous nuclear ribonucleoprotein D-like (HNRPDL), transcript variant 2
HNRNPH3	Heterogeneous nuclear ribonucleoprotein H3 isoform a variant
HNRNPA0	Heterogeneous nuclear ribonucleoprotein A0
***Ribosomal proteins***	
SF1	Splicing factor 1, isoform CRA_d
RAVER1	Ribonucleoprotein PTB-binding 1
THOC3	THO complex subunit 3
LTV1	Protein LTV1 homolog
BOP1	Ribosome biogenesis protein BOP1
SRP9	Signal recognition particle 9 kDa protein
GTPBP4	Nucleolar GTP-binding protein 1
WDR12	Ribosome biogenesis protein WDR1
RPS10	40S ribosomal protein S10
RRP9	U3 small nucleolar RNA-interacting protein
NOL6	Nucleolar protein 6
NLE1	cDNA FLJ57449, highly similar to Notchless homolog 1
RBM12B	RNA binding protein 12B
RPL28	60S ribosomal protein L28
NOP2	Isoform 2 of Probable 28S rRNA (cytosine (4447)-C (5) methyltransferase
***Actin-myosin network***	
ARPC1B	Actin related protein 2/3 complex, subunit 1B, 41 kDa
ARPC2	Arp2/3 complex 34 kDa subunit
ARPC3	Actin-related protein 2/3 complex subunit 3
MYO1C	Unconventional myosin-Ic
MYO6	Unconventional myosin-VI
ACTR2	Actin-related protein 2
ACTR3	ARP3 actin-related protein 3 homolog (Yeast)
CTTN	Cortactin isoform a variant (Fragment)
CAPZA2	Capping protein (Actin filament) muscle Z-line, alpha 2 variant
MYO1B	Unconventional myosin-Ib
***Others***	
USP11	Ubiquitin carboxyl-terminal hydrolase 11
TRIM25	Tripartite motif-containing 25, isoform CRA_a
ATXN2L	Ataxin 2-like, isoform CRA_e
HLCS	Biotin—protein ligase
RNF220	E3 ubiquitin-protein ligase RNF220
IRF2BPL	Interferon regulatory factor 2-binding protein-like
BTBD9	BTB/POZ domain-containing protein 9
ACC	Acetyl-CoA carboxylase
ACACB	Isoform 3 of Acetyl-CoA carboxylase 2
ANKHD1	Isoform 6 of Ankyrin repeat and KH domain-containing protein 1
AURKB	Aurora kinase B
CBX1	Chromobox protein homolog 1
GOLGA4	Golgin subfamily A member 4
YTHDF3	YTH domain family, member 3, isoform CRA_a
G3BP2	Ras-GTPase activating protein SH3 domain-binding protein 2
HSPH1	Similar to heat-shock protein 105 kDa
TP53	Tumor protein p53
LIMA1	Epithelial protein lost in neoplasm beta variant
CBX5	Chromobox homolog 5 (HP1 alpha homolog, Drosophila)
H2AFV	Histone H2A
BPIFA1	Isoform 2 of BPI fold-containing family A member 1

**Table 5:** Common host protein that interact with JCV LT-Ag and JCV Sm t-Ag.

Gene Code and Categories	Gene Name
***Actin-myosin network***	
ACTR3	ARP3 actin-related protein 3 homolog (Yeast), isoform
MYO6	Unconventional myosin-VI
SF1	Splicing factor 1, isoform CRA_d
CTTN; EMS1	Cortactin isoform a variant (fragment)
MYO1B	Unconventional myosin-Ib
ARPC2	Arp2/3 complex 34 kDa subunit
MYO1C	Unconventional myosin-Ic
ARPC3	Actin-related protein 2/3 complex subunit 3
CAPZA2	Capping protein (Actin filament) muscle Z-line, alpha 2 variant
ARPC1B	Actin related protein 2/3 complex, subunit 1B, 41 kDa
ACTR2	Actin-related protein 2
***Ribosomal/RNA binding proteins***	
RPL28	60S ribosomal protein L28
RAVER1	Ribonucleoprotein PTB-binding 1
RBM12B	RNA-binding protein 12B
HNRNPH3	Heterogeneous nuclear ribonucleoprotein H3 isoform a variant
RPS10	40S ribosomal protein S10
***Others***	
THOC3	THO complex subunit 3
BPIFA1	Isoform 2 of BPI fold-containing family A member 1
SLC25A3	Phosphate carrier protein, mitochondrial
G3BP2	Ras-GTPase activating protein SH3 domain-binding protein 2
HSPH1	Similar to heat-shock protein 105 kDa
TP53	Tumor protein p53
LIMA1	Epithelial protein lost in neoplasm beta variant
LTV1	Protein LTV1 homolog
CAPRIN1	Isoform 2 of Caprin−1
CBX5	Chromobox homolog 5 (HP1 alpha homolog, Drosophila)
H2AFV	Histone H2A

## Supplementary Materials

The following are available online at https://www.mdpi.com/1999-4915/12/10/1192/s1, Figure S1: A complete list of the JCV LT-Ag- and JCV Sm t-Ag-associated host proteins. Figure S2: A selected list of the JCV LT-Ag- and JCV Sm t-Ag-associated host proteins. Figure S3: A list of the common host proteins associated with both JCV LT-Ag and JCV Sm t-Ag. Figure S4: A list of the host proteins uniquely associated with JCV LT-Ag only. Figure S5: A list of the host proteins uniquely associated with JCV Sm t-Ag only. Figure S6: Analysis of the proteomics data for JCV LT-Ag using FunRich program. Figure S7: Analysis of the proteomics data for JCV Sm t-Ag using FunRich program. Figure S8: Analysis of the common proteomics data for JCVLT-Ag and JCV Sm t-Ag using FunRich program.
