# Supplementary material for: A Comprehensive Proteomics Analysis of the JC Virus (JCV) Large and Small Tumor Antigen Interacting Proteins: Large T Primarily Targets the Host Protein Complexes with V-ATPase and Ubiquitin Ligase Activities While Small t Mostly Associates with Those Having Phosphatase and Chromatin-Remodeling Functions
Source: Viruses. 2020 Oct 20;12(10):1192. doi: 10.3390/v12101192 (PMC7594058; doi:10.3390/v12101192)
Supplement: Supplementary file 1 [file viruses-12-01192-s001.zip › 3. Legends for the supplemental data. final-sent, 9-29-20.docx]

**Figure legends for the supplemental materials:**

**Figure S1.** A complete list of the JCV LT-Ag- and JCV Sm t-Ag-associated host proteins identified by the two independent AP/MS runs.

**Figure S2**. A selected list of the JCV LT-Ag- and JCV Sm t-Ag-associated host proteins identified by the two independent AP/MS runs. These interacting proteins were selected based on the criteria explained in the main text of the manuscript.

**Figure S3.** A list of the common host proteins associated with both JCV LT-Ag and JCV Sm t-Ag are identified in 1^st^ and 2^nd^ AP/MS run. These interacting proteins were selected based on the criteria stated in the main text.

**Figure S4.** A list of the host proteins uniquely associated with JCV LT-Ag only are identified in 1^st^ and 2^nd^ AP/MS runs. These interacting proteins were selected based on the criteria stated in the main text.

**Figure S5.** A list of the host proteins uniquely associated with JCV Sm t-Ag only are identified in 1^st^ and 2^nd^ AP/MS runs. These interacting proteins were selected based on the criteria stated in the main text.

**Figure S6**. Analysis of the proteomics data for JCV LT-Ag using “FunRich program, which allows us to analyze AP/MS data based on the different categories such as biological processes, biological pathways, cellular components, molecular functions, protein domains, transcription factors, site of expressions and clinical phenotypes. The host proteins targeted by the JCV LT-Ag were further analyzed by using “FunRich”, based on the p=0.05 reference value. Percentage of genes is defined as the ratio of the host proteins that interact with JCV LT-Ag or JCV Sm t-Ag to the available number of proteins present in the FunRich database in that specific category.

**Figure S7**. Analysis of the proteomics data for JCV Sm t-Ag using “FunRich program. The host proteins targeted by the JCV Sm t-Ag were further analyzed by using “FunRich”, based on the p=0.05 reference value. Percentage of genes is defined as the ratio of the host proteins that interact with JCV LT-Ag or JCV Sm t-Ag to the available number of proteins present in the FunRich database in that specific category.

**Figure S8**. Analysis of the common proteomics data for JCVLT-Ag and JCV Sm t-Ag using “FunRich program. The common host proteins targeted by the JCV LT-Ag and JCV Sm t-Ag were further analyzed by using “FunRich”, based on the p=0.05 reference value. Percentage of genes is defined as the ratio of the host proteins that interact with JCV LT-Ag or JCV Sm t-Ag to the available number of proteins present in the FunRich database in that specific category.
