# Supplementary figures and images for: A Comprehensive Proteomics Analysis of the JC Virus (JCV) Large and Small Tumor Antigen Interacting Proteins: Large T Primarily Targets the Host Protein Complexes with V-ATPase and Ubiquitin Ligase Activities While Small t Mostly Associates with Those Having Phosphatase and Chromatin-Remodeling Functions
Source: Viruses. 2020 Oct 20;12(10):1192. doi: 10.3390/v12101192 (PMC7594058; doi:10.3390/v12101192)

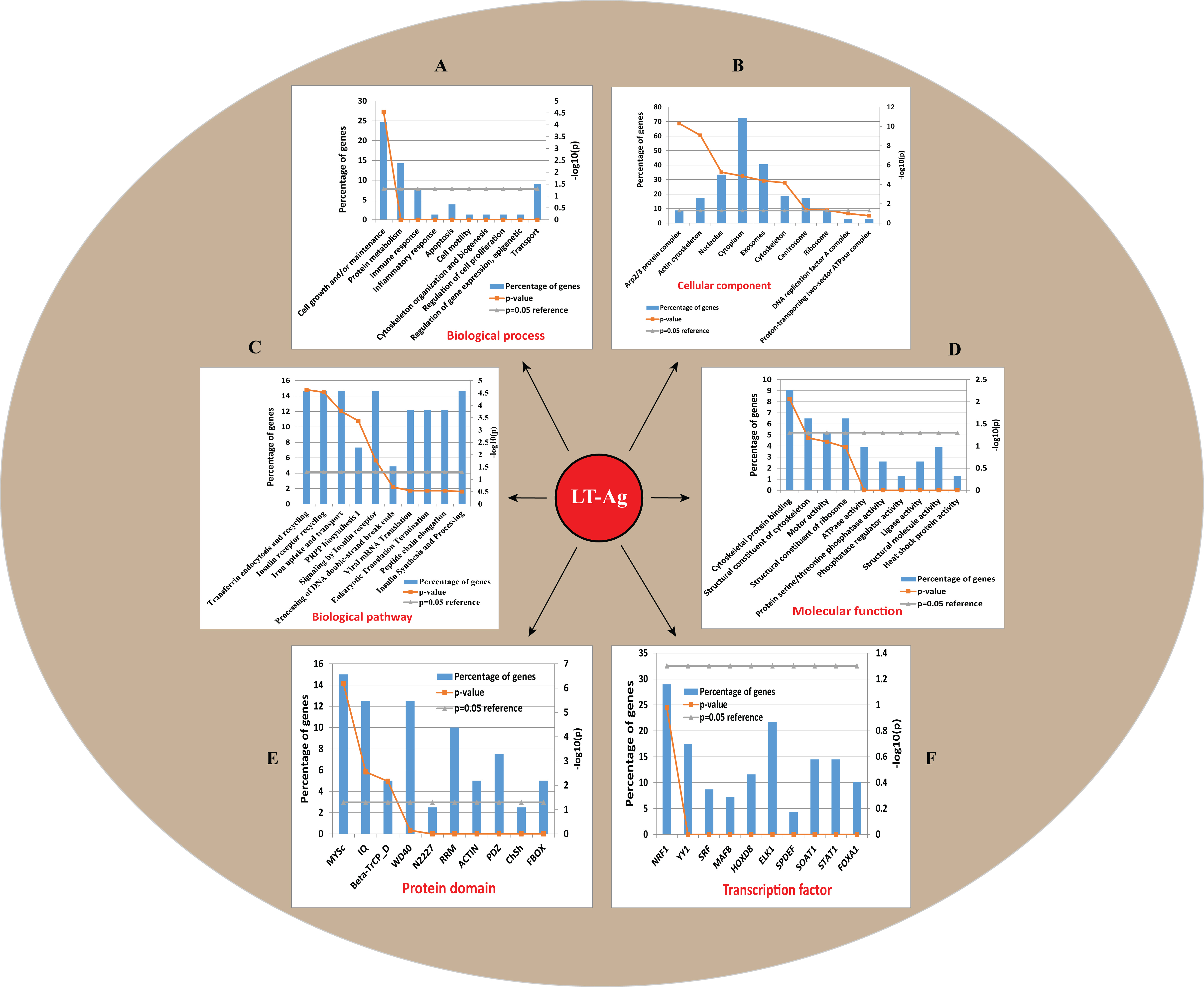

Supplement: Supplementary file 1 [file viruses-12-01192-s001.zip › Figure S6. Analysis of the proteomics data for JCV LT-Ag using FunRich program.jpg]

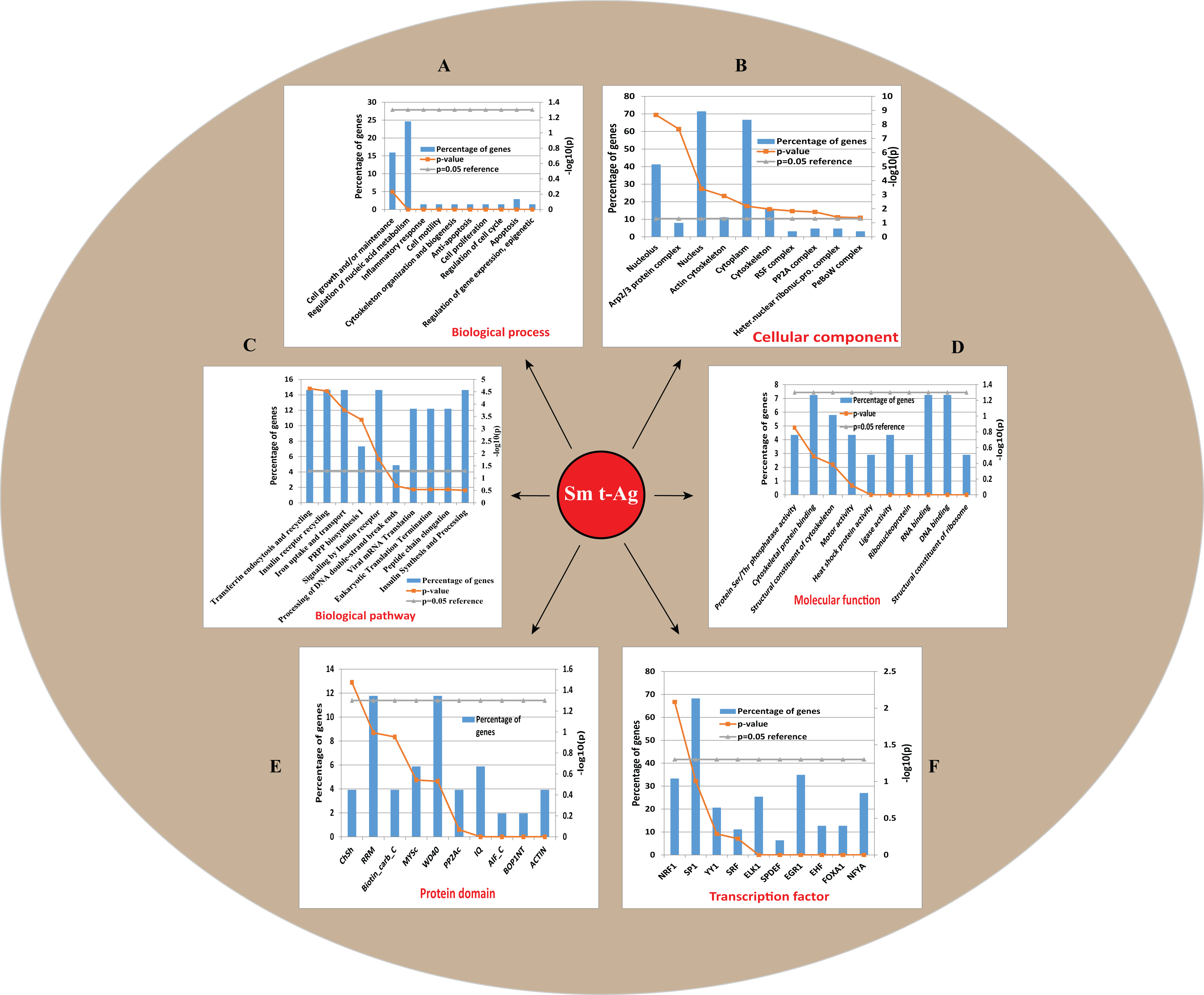

Supplement: Supplementary file 1 [file viruses-12-01192-s001.zip › Figure S7. Analysis of the proteomics data for JCV Sm t-Ag using FunRich program.jpg]

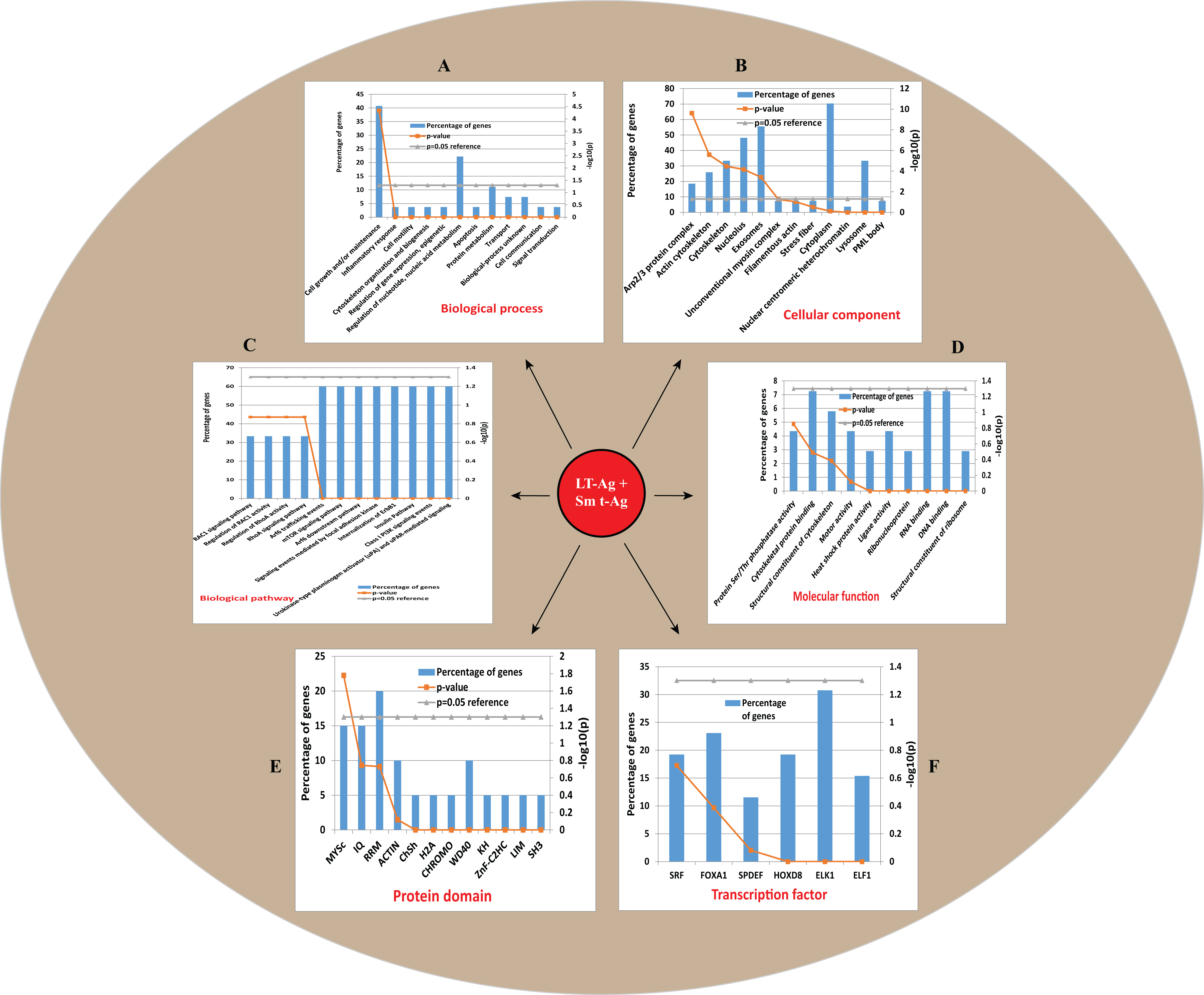

Supplement: Supplementary file 1 [file viruses-12-01192-s001.zip › Figure S8. Analysis of the common proteomics data for JCVLT-Ag and JCV Sm t-Ag using FunRich program, 8-28-20.jpg]
